# NF-κB-active tumors with matrix CAFs and suppressive immunity as key resistance mechanisms to chemoradiation in rectal cancer

**DOI:** 10.1038/s12276-026-01792-2

**Published:** 2026-08-04

**Authors:** Junseong Park, Sun Shin, Jae Woong Kim, Dokyeong Kim, Sumin Cho, Hyun Ho Kim, Changhyeok An, Sug Hyung Lee

**Affiliations:** 1 Cancer Evolution Research Center, College of Medicine, The Catholic University of Korea, Seoul, Republic of Korea; 2 Precision Medicine Research Center, College of Medicine, The Catholic University of Korea, Seoul, Republic of Korea; 3 Catholic Hematopoietic Stem Cell Bank, College of Medicine, The Catholic University of Korea, Seoul, Republic of Korea; 4 Department of Microbiology, College of Medicine, The Catholic University of Korea, Seoul, Republic of Korea; 5 Department of Medical Sciences, Graduate School of The Catholic University of Korea, Seoul, Republic of Korea; 6 Department of Pathology, College of Medicine, The Catholic University of Korea, Seoul, Republic of Korea; 7 Department of Surgery, College of Medicine, The Catholic University of Korea, Seoul, Republic of Korea

**Keywords:** Cancer therapeutic resistance, Rectal cancer, Cancer genomics, Cancer microenvironment, Chemotherapy

## Abstract

Most patients with intermediate-to-high-risk locally advanced rectal cancer (LARC) undergo neoadjuvant chemoradiotherapy (NCRT), but reliable predictive biomarkers and well-defined resistance mechanisms remain lacking. We sought biomarkers and molecular mechanisms of NCRT resistance in LARC. We analyzed paired pre-NCRT and post-NCRT biopsies from 26 patients with intermediate-to-high-risk LARC using single-cell RNA sequencing and spatial transcriptomics. Suboptimal responders showed pre-NCRT activation of NF-κB, Wnt, Notch, VEGF, fatty acid, and glutamate pathways, whereas optimal responders exhibited higher cell-cycle and oxidative phosphorylation activity in tumor cells. We experimentally validated that NF-κB inhibition increased chemoradiation sensitivity in colorectal cancer cells. Matrix cancer-associated fibroblasts (mCAFs), characterized by transforming growth factor-β/extracellular matrix programs, preferentially interacted with NF-κB-high tumor cells and immunosuppressive populations in suboptimal responders, whereas optimal responders exhibited cytotoxic CD8⁺ T cell expansion and conventional dendritic cell-2 enrichment. Spatial mapping revealed mCAF-dense, extracellular matrix-rich niches colocalized with NF-κB-rich tumor-adjacent regions in suboptimal cases. Synchronous rectal and sigmoid tumors from one patient shared suboptimal response with NF-κB activation and mCAF enrichment, suggesting the resistance arises from adaptive epithelial-stromal programs rather than site-specific genetics. Collectively, integrated multicompartment signatures, rather than a single molecular profile, define an NCRT-resistant ecosystem in which tumor NF-κB activation, mCAF enrichment, and immune suppression converge to sustain non-response. In line with this framework, combined NF-κB and oxidative phosphorylation scores stratified response status with robust performance (area under the curve = 0.875) and highlighted resistance-associated therapeutic targets.

## NF-κB activation drives resistance in rectal cancer treatment

Rectal cancer, a significant global health concern, often requires neoadjuvant chemoradiotherapy (NCRT) for locally advanced cases. However, only a third of patients achieve complete pathological response, highlighting the need for predictive biomarkers. This study uses single-cell RNA sequencing and spatial transcriptomics to explore tumor and tumor microenvironment dynamics in response to NCRT. Researchers analyzed serial biopsies from 26 patients, identifying two tumor cell subpopulations with distinct signaling pathways. Tumor_2, associated with resistance, showed elevated NF-κB, Wnt, and VEGF pathways. The study also identified cancer-associated fibroblasts contributing to resistance through extracellular matrix remodeling and transforming growth factor-β signaling. These findings suggest that targeting NF-κB and cancer-associated fibroblast interactions could enhance NCRT efficacy. Future research may focus on integrating these biomarkers into clinical practice to improve treatment outcomes for patients with intermediate-to-high-risk rectal cancer. This summary was initially drafted using artificial intelligence, then revised and fact-checked by the author.

## Introduction

Rectal cancer is the eighth most common malignancy worldwide, accounting for approximately one-third of all colorectal cancer cancers^1^. For most patients with locally advanced rectal cancer (LARC), defined as T3-4 with node-negative and any T with node-positive, neoadjuvant chemoradiotherapy (NCRT) followed by radical proctectomy remains the standard of care. This approach achieves effective local control, sphincter preservation, and acceptable toxicity profiles^2,3^. Recent randomized trials for LARCs with low-to-intermediate risk have shown that neoadjuvant chemotherapy alone can achieve local recurrence and survival outcomes comparable to NCRT^4,5^; however, patients with moderate-to-high risk still require NCRT. Despite the advances, only about one-third of patients with LARC treated with NCRT achieve a pathological complete response^6,7^. However, no robust clinical, pathological, radiological, or molecular biomarkers currently exist to predict therapeutic responsiveness^8^, underscoring the need to elucidate mechanisms of resistance and to identify predictive markers of NCRT response in LARC.

Cancer progression and therapy response are influenced not only by tumor cells but also by the tumor microenvironment (TME), which comprises normal epithelial cells, fibroblasts, vascular endothelial cells, immune, and inflammatory cells^9–12^. Therapeutic interventions such as chemotherapy and radiotherapy can modify both tumor and TME components, with effects that may be either tumor-suppressive or tumor-promoting^13,14^. For instance, chemoradiation activates diverse signaling pathways within tumors, generating complex immune responses^15^. Radiation further alters multiple cellular components of the TME^16^, influencing revascularization, inflammation, immune modulation, fibroblast activation, and extracellular matrix (ECM) remodeling. These findings highlight the importance of simultaneously interrogating tumor and TME cells during NCRT to understand the mechanisms of treatment response and resistance, as well as the co-evolution of tumor and TME compartments in LARC.

Serial, pairwise tumor sampling before and after neoadjuvant therapy is essential to capture the dynamic biology of treatment response^12,17,18^. However, most existing transcriptomic studies in LARC rely on post-treatment samples or bulk sequencing rather than serial, single-cell analyses^19,20^. In other cancers, such as esophageal carcinoma, single-cell RNA sequencing (scRNA-seq) has revealed treatment-induced alterations in both tumor and TME compartments during NCRT^21^. A recent scRNA-seq study of low-to-intermediate-risk LARCs treated with neoadjuvant chemotherapy demonstrated therapy-induced remodeling dominated by cancer-associated fibroblasts (CAFs), which can promote either immune activation or epithelial–mesenchymal transition-driven resistance^22^. However, no comparable single-cell studies have systematically analyzed serial samples before and after NCRT in intermediate-to-high-risk LARCs.

In this study, we aimed to characterize the molecular features of both tumor and TME cells in LARC treated with NCRT. Using serial tumor biopsies and matched non-tumor mucosae, we applied scRNA-seq and spatial-seq (i) to delineate the cellular and molecular mechanisms underlying NCRT resistance and (ii) to identify predictive biomarkers that can stratify patients according to therapeutic responsiveness. Our analyses further sought to integrate tumor-intrinsic signaling programs, stromal remodeling, and immune reprogramming into a unified framework for response prediction in rectal cancer.

## Materials and methods

### Human samples and clinical information

**Fig. 1:**
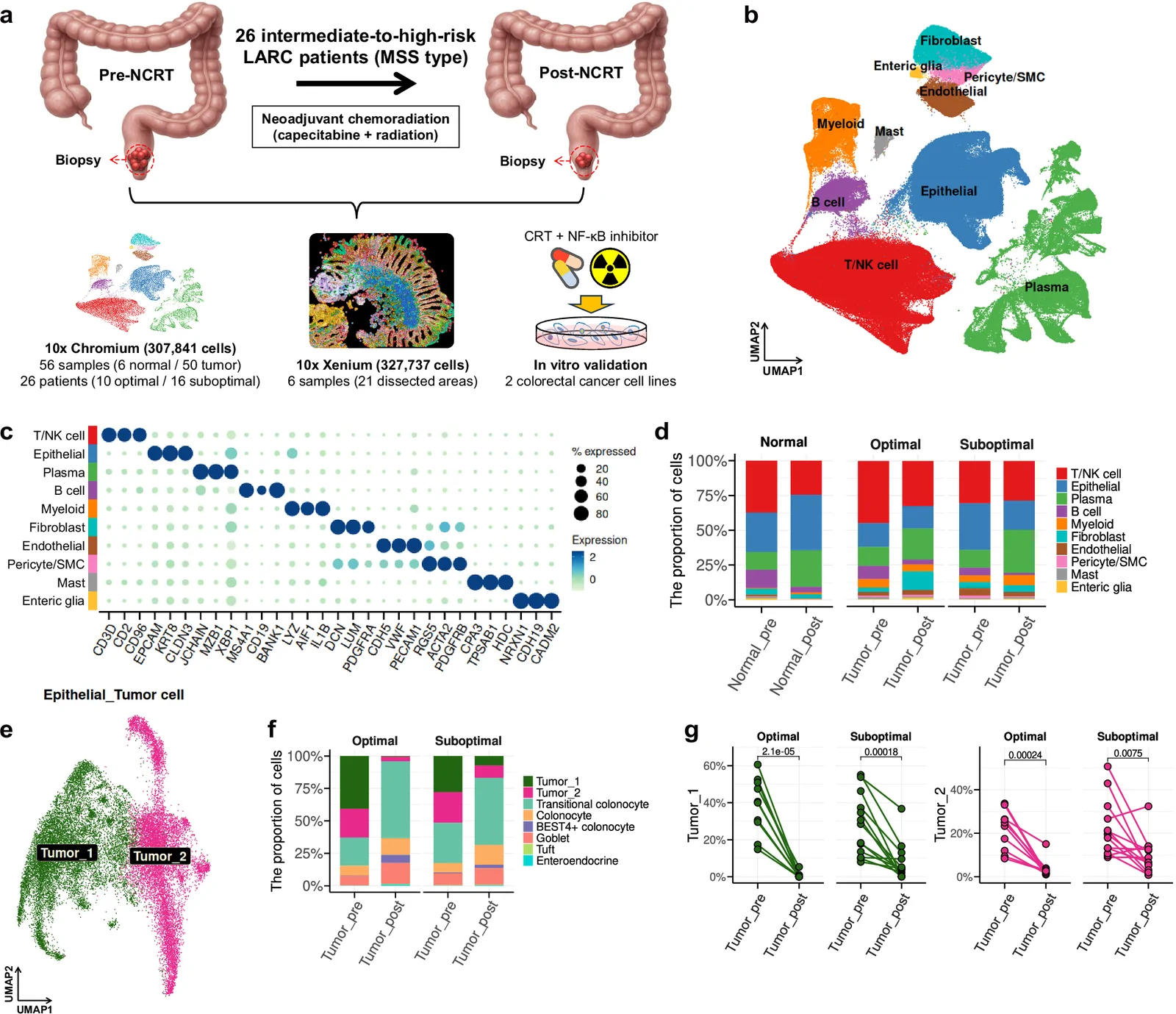
NCRT-driven remodeling of the LARC ecosystem with response group-specific patterns. **a** Study design and sampling scheme. **b** UMAP embedding of all single-cell RNA-seq data colored by major cell lineages. **c** Dot plot of canonical marker genes used for cluster annotation across epithelial, immune, stromal, and vascular compartments. **d** Stacked bar plots showing the proportion of major cell types across groups. **e** UMAP plot of the re-clustered tumor cell compartment colored by subpopulations. **f** Bar plots comparing the distribution of epithelial subclusters according to treatment status and response groups. **g** Within-patient changes in tumor subcluster proportions according to treatment status and response groups. Each line connects paired samples from the same patients. Statistical significance was assessed using paired *t* test. LARC, locally advanced rectal cancer; MSS, microsatellite-stable; NCRT, neoadjuvant chemoradiotherapy; NK, natural killer; SMC, smooth muscle cell; UMAP, uniform manifold approximation and projection.

Biopsy samples were obtained from 26 patients with LARC who received NCRT. Of the patients initially enrolled, no participants withdrew; demographic and clinical variables, including age, sex, clinical stage, and treatment details, were summarized in Supplementary Table 1. All procedures were conducted in accordance with the Declaration of Helsinki, with written informed consent from all participants, and approved by the Institutional Review Board of The Catholic University of Korea, Bucheon St Mary’s Hospital (IRB number: HC20TNSI0076). All LARCs analyzed were located in the lower or middle third of the rectum, within 10 cm away from the anal verge. Risk stratification (low, intermediate, high) followed the European Society for Medical Oncology classification^23^. Endoscopic biopsies were collected from both tumor tissues and matched normal mucosa at least 10 cm from the tumor margin. On the basis of pathological tumor regression grading^24^, patients were categorized as optimal responders (grade 0, 1; *n* = 10) or suboptimal responders (grade 2, 3; *n* = 16). Tumor biopsies were obtained before NCRT (pre-CRT) and after NCRT (post-CRT) (Fig. 1a). For scRNA-seq, tumor and normal samples from each patient were analyzed separately. For Xenium spatial transcriptomics (10× Genomics), tumor and normal specimens were embedded in a single block and analyzed together. The preoperative NCRT regimen consisted of oral capecitabine chemotherapy combined with fractionated pelvic radiotherapy delivering a total dose of 50.4 Gy over 5–6 weeks^25^.

### Tissue dissociation and scRNA-seq data processing

Tissue dissociation was conducted using the Human Tumor Dissociation Kit (Miltenyi Biotec) according to the manufacturer’s protocol. In brief, freshly minced tissue was enzymatically digested using kit reagents on gentle MACS Dissociator (Miltenyi Biotec). After homogenization, tissue suspensions were filtered through a 70-μm MACS SmartStrainer (Miltenyi Biotec), and single cells within the supernatant were washed with phosphate-buffered saline (PBS; HyClone). Cells were then resuspended in RPMI-1640 medium supplemented with 10% fetal bovine serum (HyClone), and cell counts and viability were assessed using Countess Automated Cell Counter (ThermoFisher Scientific). The resulting single-cell suspensions were then used to generate scRNA-seq libraries with Chromium Next GEM Single Cell 3ʹ v3.1 kit (10× Genomics), following the manufacturer’s instructions, and sequenced using Illumina NovaSeq platform. Sequencing reads were aligned to the human reference genome (GRCh38) and processed with Cell Ranger 7.1.0 pipeline (10× Genomics). After doublet removal using DoubletFinder^26^, low-quality cells with fewer than 1,500 unique molecular identifiers or greater than 20% mitochondrial genes were excluded (Supplementary Fig. 1a–d). All individual datasets were then integrated with batch correction using RPCA algorithm. Subsequent analyses, including normalization (SCTransform), uniform manifold approximation and projection (UMAP) dimensionality reduction, and differentially expressed gene (DEG) analyses, were performed in R (v4.4.2) using Seurat (v5.3.0)^27^, as described previously^28,29^. Clusters were manually annotated by comparing canonical marker genes and DEGs for each cluster.

### Advanced analyses of scRNA-seq data

Inferred single-cell copy-number alteration (CNA) profiles were generated using inferCNV (v1.22.0). InferCNV was applied to all epithelial cells using goblet cells as the reference population. Chromosome-level CNA signals for chromosome 8 alteration, chromosome 13 gain, chromosome 14 loss, and chromosome 20 gain were extracted from the inferCNV output, added to the Seurat metadata, and used for dimensional reduction and re-clustering of epithelial cells. A distinct cluster showing concordant deviations across these CNA features was annotated as malignant. No arbitrary per-cell cut-off was applied; malignancy was defined at the cluster level and confirmed by concordance with the inferCNV heatmap. Receiver operating characteristic (ROC) curves with area under the curve (AUC) were computed using the R package pROC (v1.19.0.1). Cell–cell interactions were analyzed using CellChat (v2.2.0)^30^ and NicheNet (v2.2.1)^31^. Epithelial cells were annotated for intrinsic-consensus molecular subtypes (iCMSs)^32^, and per-cell enrichment scores were used to assign relative iCMS states. Pseudotime trajectories of tumor cells were inferred using Monocle3 (v1.3.7)^33^, and RNA velocity was estimated from spliced and unspliced transcript counts using velocyto^34^.

### Enrichment analyses and module scoring

All enrichment analyses and module scoring were performed using UCell (v2.10.1)^35^. For pathway enrichment, we used gene sets from the c2 canonical pathways collection in MSigDB^36^. Custom gene signatures were defined as follows. For the NF-κB score, we used: *NFKB1*, *NFKBIB*, *TNF*, *TNFRSF6B*, *TNFRSF10A*, *TNFRSF10B*, *TNFRSF10D*, *TNFAIP2*, *IL1B*, *TRAF2*, *TRAF4*, *TRAF7*, *TAB2*, *TAB3*, and *C3*. For the oxidative phosphorylation (OxPhos) score^37^, we used: *LDHB, LDHA, COX5A, PGK1, MZT2A, MZT2B, NDUFAB1, NDUFB3, ATP5PD,* and *PKM*. For the matrix CAF (mCAF) score^38^, we used: *INHBA, FAP, MMP1, MMP3, MMP12, COL5A2, COL6A3, COL8A1, TNC, TGFBI, TGFBR1, ADAM12,* and *ADAMTS12*. For the cytotoxicity score, we used: *CD8A, CD8B, GZMA, GZMB, GZMK, GZMH, GZMM, PRF1, NKG7,* and *IFNG*. For composite score analysis, NF-κB and OxPhos scores were first *z*-score-standardized across samples and then combined with equal weighting as *z*(NF-κB score) − *z*(OxPhos score).

### Spatially resolved transcriptome profiling

Fresh frozen rectal cancer tissues were processed for in situ spatial transcriptomics using the Xenium in situ platform (10× Genomics), according to the manufacturer’s instructions. Briefly, representative tumor-containing blocks were selected by a pathologist, and sections were mounted onto Xenium slides. Tissue sections were incubated on a thermocycler for probe hybridization, ligation, and amplification with a custom gene panel targeting 475 genes (Supplementary Table 2). Automated cell segmentation and per-cell transcript calling were carried out using the Xenium Onboard Analysis pipeline, yielding cell-level count matrices and associated cell centroid coordinates. For quality control, cells with fewer than 10 transcripts were excluded from downstream analyses, and the remaining cells were processed using the Seurat pipeline, including normalization, UMAP dimensionality reduction, DEG analysis, and spatial niche analysis. Tissue images overlaid with gene expression were generated using Xenium Explorer (v4.1.0). Proximity analyses based on intercellular distance were performed using the R packages sf (v1.0.21) and nngeo (v0.4.8). To reduce the effect of occasional cell-type misannotation, spatial proximity to a queried cell type was considered valid only when at least five cells of that type were present within the local neighborhood; when this criterion was met, the distance to the nearest such cell was recorded. Fibroblasts located within 30 μm of malignant epithelial cells were classified as tumor-adjacent, whereas those located ≥150 μm away were classified as tumor-distant.

### Cell culture and in vitro chemoradiation

Two human colorectal cancer cell lines with microsatellite-stable (MSS) type, HT29 and SW620, were cultured in RPMI-1640 (HyClone) supplemented with 10% fetal bovine serum and 1% penicillin–streptomycin (Sigma-Aldrich). Cells were maintained in a controlled environment at 37 °C in a humidified atmosphere containing 5% CO_2_, and the culture medium was replaced every 2–3 days. Mycoplasma contamination was routinely monitored by PCR and only mycoplasma-negative cells were used. For subculturing, cells were detached using 0.25% trypsin (HyClone) and neutralized with serum-containing medium. For chemoradiation, cells were pretreated with capecitabine (10 µM; Selleckchem) in the presence or absence of NF-κB inhibitor Bay11-7085 (5 µM; MedChemExpress) for 24 h, followed by irradiation at a dose of 4 Gy using a γ-irradiator (MDS Nordion). Immediately after irradiation, the medium was replaced with drug-free medium. Untreated controls underwent identical handling procedures, including medium replacement at the corresponding time points of drug treatment and irradiation.

### Cell viability assay

Cell viability was assessed using water-soluble tetrazolium assays with the Cell Counting Kit-8 (Dojindo Molecular Technologies). Cells were seeded in 96-well plates (SPL Life Sciences) at a density of 8 × 10^3^ cells per well. After the indicated treatments for 72 h, CCK-8 solution was added to each well at a final concentration of 10% (v/v) of culture medium, and cells were incubated for an additional 1.5 h at 37 °C. Absorbance at 450 nm was measured using a microplate reader (BioTek Instruments).

### Immunocytochemistry

Cells were seeded on 4-well chamber slides (SPL Life Sciences) and subjected to the indicated treatment. Samples were fixed with 4% paraformaldehyde (Biosesang) for 10 min at room temperature, permeabilized with 0.5% Triton X-100 (Amresco) in PBS for 10 min, and blocked with 5% bovine serum albumin (BioWORLD) in PBS for 1 h. Cells were then incubated overnight at 4 °C with primary antibody against NF-κB p65 (1:200; Santa Cruz Biotechnology), followed by incubation with Alexa Fluor 488-conjugated secondary antibody (1:1,000; Invitrogen) for 1 h at room temperature in the dark. Nuclei were counterstained with Hoechst (Invitrogen) for 10 min. Fluorescence images were acquired using FV3000 confocal laser scanning microscope (Olympus).

## Results

### Cellular atlas of rectal cancers in response to chemoradiation

We constructed a longitudinal single-cell atlas of 26 patients with LARC exhibiting MSS phenotypes, encompassing paired pre-CRT and post-CRT analyses. It allowed for quantitative assessment of how NCRT remodeled both the tumor and its microenvironment and how these dynamics differed according to clinical outcomes (10 optimal and 16 suboptimal cases). One patient (case 11) presented with synchronous double primary tumors located in the sigmoid colon (11S; *APC* and *TP53* mutations) and in the rectum (11R; *APC*, *FBXW7*, and *AKT1* mutations); both exhibited suboptimal responses. Paired endoscopic biopsies obtained before and after NCRT, together with matched non-tumor mucosae, were processed for scRNA-seq and integrated through a uniform computational pipeline (Fig. 1a). Unsupervised clustering resolved all major cell compartments, including epithelial, stromal, immune, and endothelial populations (Fig. 1b,c and Supplementary Fig. 1e).

Tumor samples displayed distinct cellular compositions compared with normal mucosae, characterized by higher proportions of myeloid and endothelial clusters (Supplementary Fig. 1f,g). Before treatment, the optimal group displayed significantly higher fractions of T/natural killer (NK) cells, and lower fractions of epithelial cells and fibroblasts, than the suboptimal group (Fig. 1d). Further clustering of epithelial, fibroblast, T/NK cell, B cell, plasma, and myeloid clusters is demonstrated in Supplementary Fig. 2. The overall shifts in cellular composition and patient-level paired plots for all major lineages across treatment and response groups are illustrated in Supplementary Fig. 3. Notably, within the humoral compartment, the suboptimal group showed a post-treatment increase in plasma cells together with a decrease in B cells, whereas no significant shift was observed in the optimal group, suggesting response-associated differences in humoral immune remodeling (Supplementary Fig. 3c).

### Tumor signaling states according to chemoradiation responsiveness

**Fig. 2:**
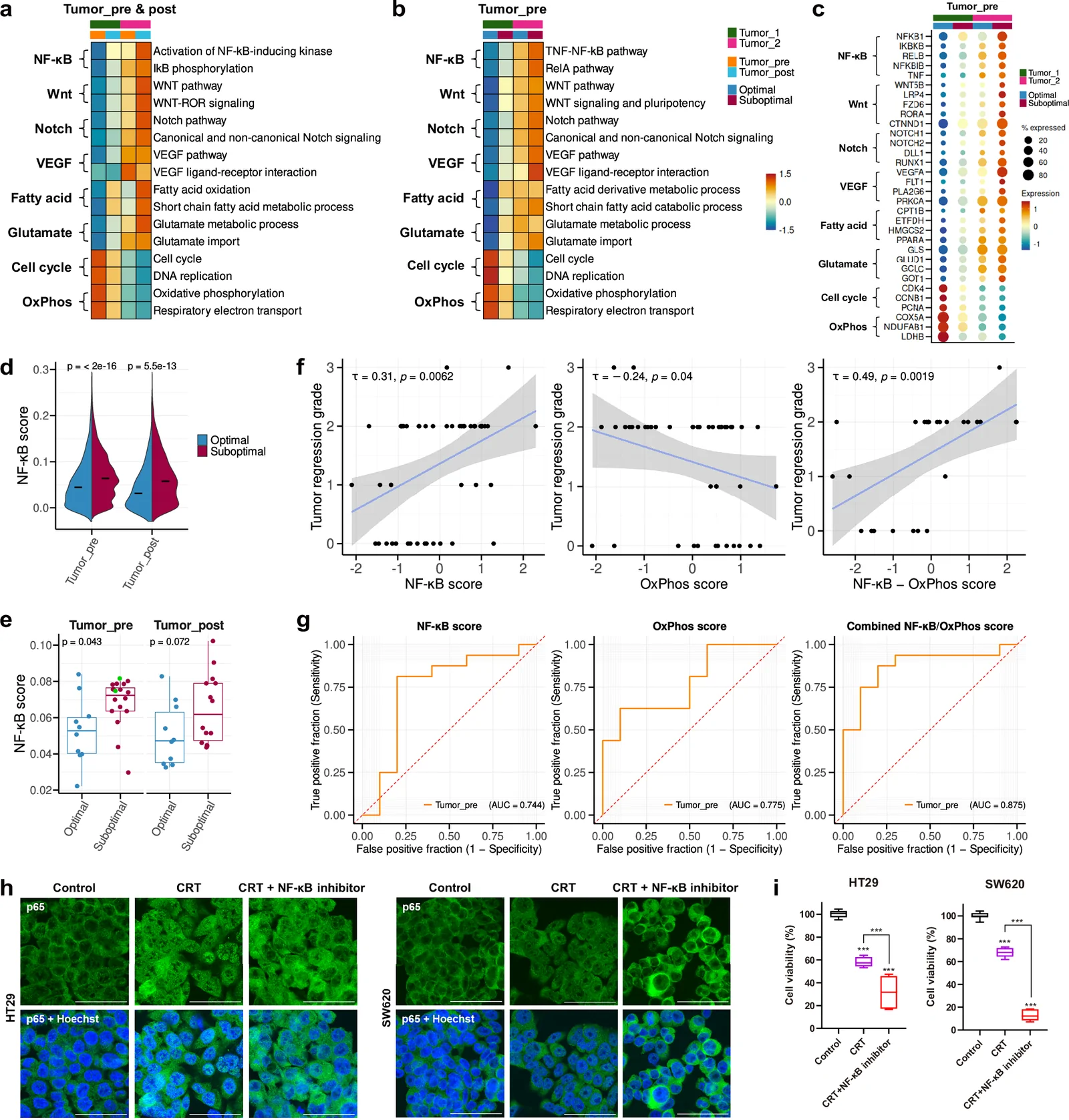
Functional annotation of tumor subclusters and experimental validation. Heatmaps showing enrichment scores for canonical pathways across tumor subclusters by treatment groups (part **a**) and response groups (part **b**). Both response groups (optimal and suboptimal) are included in part **a**. **c** Dot plot showing the average expression of representative pathway-associated genes across tumor subclusters and response groups. **d** Violin plots of single-cell NF-κB scores with median values denoted by black crossbars. **e** Sample-level NF-κB scores with each dot representing an individual sample; the synchronous primary tumors (11S and 11R) are highlighted in green. **f** Scatter plot showing the correlations of NF-κB (left), oxidative phosphorylation (OxPhos) (center), and combined scores (right) with tumor regression grade. Each dot represents a single sample, and Kendall’s tau correlation coefficient with corresponding *P*-value is annotated. **g** Receiver operating characteristic curves showing the predictive performance of NF-κB (left), OxPhos (center), and combined scores (right; NF-κB score – OxPhos score) in distinguishing optimal from suboptimal responders. The area under the curve (AUC) is indicated for each model. **h** Immunofluorescence images of the intracellular localization of NF-κB p65 (green) in HT29 and SW620 colorectal cancer cells. Cells were pretreated with capecitabine (10 µM) for 24 h with or without the NF-κB inhibitor Bay11-7085 (5 µM), followed by 4 Gy irradiation. After 72 h incubation, cells were subjected to immunocytochemistry; nuclei were counterstained with Hoechst (blue). **i** Cell viability under the same treatment conditions as in part **h** assessed using water-soluble tetrazolium assays. For parts **a**–**c**, values were *z*-score-transformed. Statistical significance was determined using Welch’s *t* test for parts **d**, **e** and one-way analysis of variance with Tukey’s post hoc test for part **i** (***P < 0.001; asterisks over treatment groups denote comparisons relative to controls). CRT, chemoradiotherapy.

To investigate transcriptional programs associated with NCRT response, we refined the malignant epithelial compartment using CNAs on chromosomes 8, 13, 14, and 20 (Supplementary Fig. 4). This analysis revealed two transcriptionally distinct tumor cell subpopulations, labeled *Tumor_1* and *Tumor_2*, identified through tumor-specific UMAP projections (Fig. 1e). In the optimal-response group, *Tumor_1* showed a significantly greater reduction after NCRT compared with *Tumor_2*. By contrast, both subpopulations persisted at comparable levels in the suboptimal group (Fig. 1f). These observations were further supported by within-patient trajectory analyses (Fig. 1g). Pathway analysis revealed that *Tumor_2*, the less-susceptible cells, exhibited higher expressions of signaling pathways related to NF-κB, Wnt, Notch, VEGF, fatty acid, and glutamate metabolism compared with *Tumor_1* (Fig. 2a). Notably, these pathways were upregulated following NCRT, suggesting that resistant tumor cells reinforced these signaling programs (Fig. 2a). Even before treatment, the suboptimal group displayed higher activity of these pathways than the optimal group in both *Tumor_1* and *Tumor_2*. Conversely, cell-cycle and OxPhos pathways, which are key pharmacodynamic targets of chemoradiation, were more active in the optimal group during the pre-CRT phase (Fig. 2b). The expression of representative genes within these pathways supported this pattern (Fig. 2c). Together, these findings suggest that the activation of cell-cycle and OxPhos programs may correlate with therapeutic responsiveness, whereas elevated levels of NF-κB, Wnt, Notch, VEGF, fatty acid, and glutamate metabolism signaling may indicate unresponsiveness. These pathways could potentially serve as predictive markers of NCRT outcomes. Mapping of epithelial cells onto iCMS programs further supported the biological distinction between the tumor subclusters. Compared with *Tumor_1*, *Tumor_2* showed lower iCMS2 and higher iCMS3 proportions. A similar shift, marked by reduced iCMS2 and increased iCMS3, was observed in post-CRT samples and in suboptimal responders (Supplementary Fig. 5a–c), indicating that the identified tumor states are anchored in established colorectal cancer epithelial programs while capturing treatment-associated and response-associated changes. In parallel, pseudotime increased from *Tumor_1* to *Tumor_2*, and RNA velocity was substantially more pronounced in the optimal group than in the suboptimal group, suggesting more plastic state transitions in responsive tumors, whereas suboptimal tumors represent less dynamically perturbed program under treatment pressure (Supplementary Fig. 5d,e).

Given the pleiotropic downstream effects of NF-κB, we next explored the predictive relevance of NF-κB and OxPhos programs in NCRT resistance. The NF-κB activity score was significantly higher in the suboptimal compared with the optimal group both before and after NCRT (Fig. 2d,e), and this score was inversely correlated with pathological tumor regression grade (Fig. 2f), suggesting that enhanced NF-κB activity contributes to poor tumor regression. OxPhos scores showed a positive correlation, and the combined NF-κB/OxPhos score exhibited the strongest overall correlation with the tumor regression grade (Fig. 2f). Notably, the two synchronous primary tumors obtained from one patient (11S and 11R) displayed high NF-κB scores (Fig. 2e). As a classifier, the NF-κB and OxPhos scores effectively distinguished between optimal and suboptimal responders, showing strong predictive performance at baseline; combined NF-κB and OxPhos scores showed improved performance (AUC = 0.875; Fig. 2g). To functionally validate the role of NF-κB in resistance, we performed in vitro chemoradiation assays using two MSS colorectal cancer cell lines, HT29 and SW620. CRT induced 30–50% cell death, whereas pharmacological inhibition of NF-κB by the Bay 11-7085 markedly sensitized cells to treatment and further increased CRT-induced cytotoxicity (Fig. 2h,i). These results confirmed that NF-κB activation promoted chemoradiation unresponsiveness in colorectal cancer.

### CAF subpopulations related to chemoradiation unresponsiveness

**Fig. 3:**
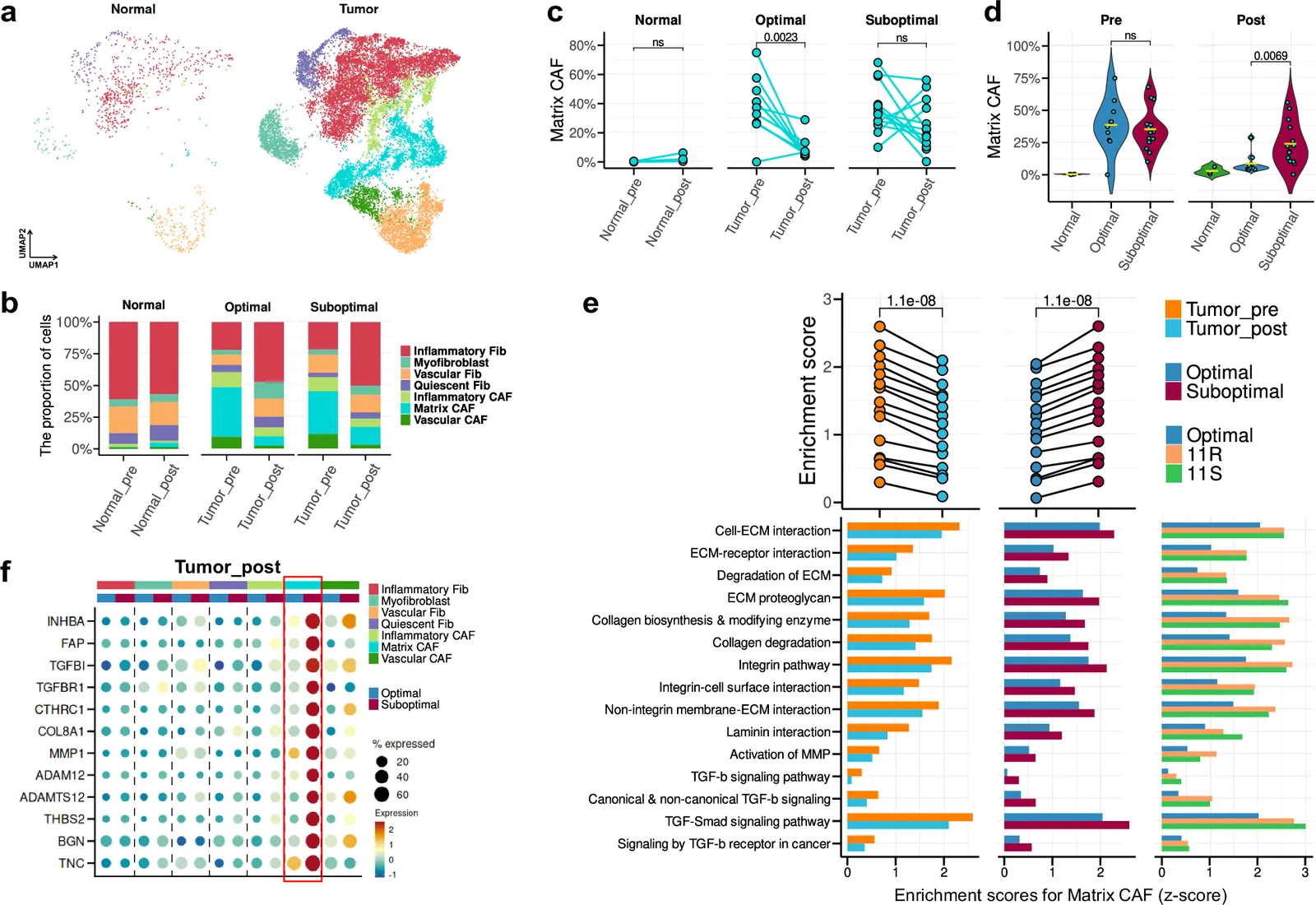
Fibroblast subsets associated with therapeutic responses to neoadjuvant chemoradiotherapy. **a** Uniform manifold approximation and projection (UMAP) visualization of fibroblast subclusters from normal and tumor samples, showing distinct lineage structures across conditions. **b** Stacked bar plots illustrating the distribution of fibroblast subclusters across groups. **c**,**d** Comparisons of matrix cancer-associated fibroblast (mCAF) proportions across treatment status and response groups. In part **c** paired pre-chemoradiotherapy (CRT) and post-CRT samples from the same patients are connected by lines; in part **d** each dot represents an individual sample. Statistical significance was assessed using paired *t* test (part **c**) and Welch’s *t* test (part **d**). **e** Pathway enrichment analysis of mCAFs comparing pretreatment and post-treatment samples (left; optimal and suboptimal combined) and optimal versus suboptimal responders (center; treatment groups combined). Each dot represents a gene set, and statistical significance for each comparison was assessed using paired *t* test (top). Pathway enrichment profiles of optimal responders were separately compared with the two synchronous primary tumors, 11S and 11R (right). **f** Dot plot showing the average expression of canonical mCAF genes in the post-CRT phase across fibroblast subclusters and response groups. Values were z-score-transformed; mCAF cluster is highlighted with red rectangle. ECM, extracellular matrix; fib, fibroblast; MMP, matrix metalloproteinase; ns, not significant; TGF-β, transforming growth factor-beta.

**Fig. 4:**
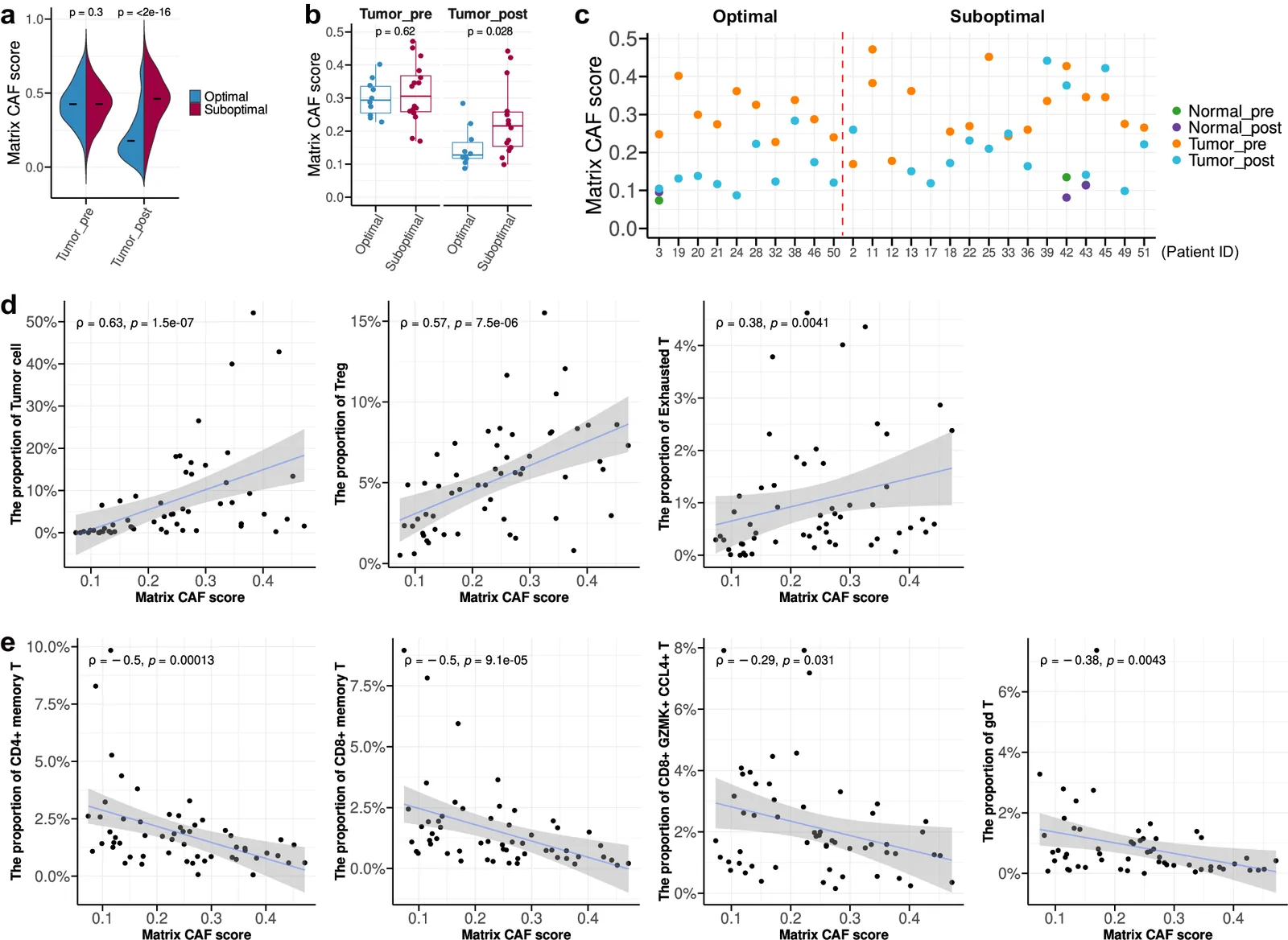
Quantitative mCAF axis of neoadjuvant chemoradiotherapy response. **a** Violin plots showing single-cell matrix cancer-associated fibroblast (mCAF) scores, with median values marked by black crossbars. **b** Sample-level mCAF scores, with each dot indicating an individual sample; for parts **a**, **b**, statistical significance was determined using Welch’s *t* test. **c** Patient-level mCAF scores plotted across treatment and response groups. Each dot represents a sample, and the red dashed line marks the boundary separating optimal and suboptimal responder groups. **d**,**e** Scatter plots depicting correlations between mCAF scores and the proportion of selected subclusters. Each dot corresponds to an individual sample, and Spearman’s rank correlation coefficients (*ρ*) with associated *P*-values are displayed. Shaded regions represent 95% confidence intervals. T_reg_, regulatory T.

The subclustering of fibroblasts revealed distinct cell states of CAFs (Supplementary Fig. 2b), which were markedly enriched in tumor samples compared with normal mucosa (Fig. 3a,b). Paired within-patient comparisons showed steeper on-treatment declines of mCAF in the optimal group than in the suboptimal group (Fig. 3c,d). Functional annotation of the mCAFs highlighted a strong enrichment for ECM organization and transforming growth factor-beta (TGF)-β signaling pathways, which were significantly reduced after NCRT but remained elevated in the suboptimal group (Fig. 3e). The synchronous primary tumors also exhibited consistent enrichment of TGF-β-related signatures in their mCAFs (Fig. 3e, right). In the post-CRT phase, canonical mCAF genes, such as *INHBA*, *FAP*, *TGFBI*, *MMP1*, and *COL8A1*, were upregulated in the suboptimal group (Fig. 3f), indicating that ECM-remodeling programs were more enriched in resistant tumors. mCAF scores were significantly higher in the suboptimal group compared with the optimal group after NCRT (Fig. 4a,b), a trend confirmed by patient-level trajectory analyses (Fig. 4c). mCAF scores correlated positively with the proportions of tumor cells, regulatory T (T_reg_), and exhausted T cells (Fig. 4d), whereas scores correlated negatively with CD4⁺ memory, CD8⁺ memory, CD8⁺ GZMK⁺ CCL4⁺, and γδ T cell subsets (Fig. 4e). This suggests that mCAFs have a pro-tumoral role in response to NCRT.

### CAF–tumor and CAF–immune crosstalk driving NCRT resistance

**Fig. 5:**
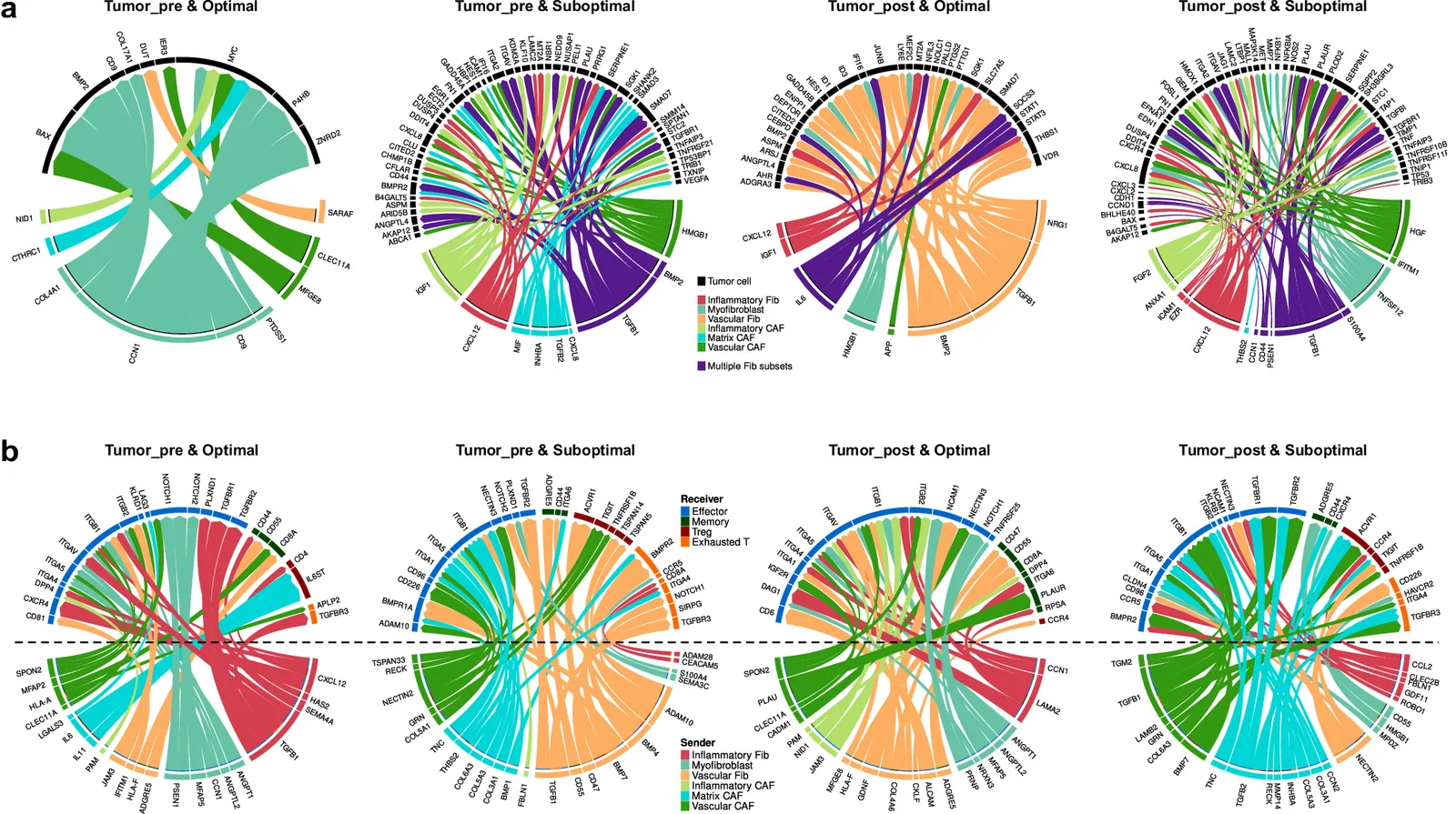
Cell–cell interactions from fibroblast subclusters to tumor and T/NK cells. **a** Circle plots illustrating inferred communication from ligands of fibroblast subclusters to target genes in tumor cells across treatment and response groups. **b** Circle plots depicting inferred communication from ligands of fibroblast subclusters to receptors on T/NK cell subclusters across treatment and response groups. For clarity, CD4^+^ helper T, CD8^+^ GZMK^+^ T, CD8^+^ GZMK^+^ CCL4^+^ T, CD8^+^ GZMB^+^ T, γδ T, and NK cells were grouped as effector; CD4^+^ memory T and CD8^+^ memory T cells were grouped as memory. Black dashed line marks the boundary separating sender fibroblast subsets and receiver immune subsets. CAF, cancer-associated fibroblast; fib, fibroblast; NK, natural killer; T_reg_, regulatory T.

We analyzed cell–cell interactions among the subclusters and found that fibroblasts were predicted to be the predominant signal senders to tumor cells in LARCs (Supplementary Fig. 6a,b). Focussing on ligand–target communication from fibroblast subclusters to tumor cells, the overall interactions in both pre-CRT and post-CRT phases were more frequent in the suboptimal group than in the optimal group, indicating an important role for fibroblast–tumor interactions in the NCRT outcomes (Fig. 5a). In the optimal group, tumor-directed signals were dominated by non-CAF fibroblast populations (myofibroblasts and vascular fibroblasts), whereas in the suboptimal group, CAFs, including mCAF, were the predominant source of signals. Specifically, several CAF-derived ligands, such as *INHBA* and *TGFB1*, converged on tumor targets related to ECM remodeling in the suboptimal cases (Fig. 5a). Notably, CAF signaling was inferred to induce tumor NF-κB in the suboptimal group, whereas this induction was absent or substantially attenuated in the optimal group (Fig. 5a). Furthermore, when the analysis was restricted specifically to the mCAF–tumor axis at baseline, mCAF-derived ligand–tumor target pairs were scarce in the optimal group but markedly enriched in the suboptimal group (Supplementary Fig. 6c), suggesting that poor responders harbor a pre-existing mCAF-driven signaling network linked to tumor programs before treatment. For fibroblast and immune cell communication, mCAF to T/NK cell interactions were more frequent in the suboptimal group in both pre-CRT and post-CRT phases (Fig. 5b). Fibroblast-derived signals to T_reg_ and exhausted T cells were also common in the suboptimal group, but were markedly reduced in the optimal group. Overall, immunosuppressive and exhaustion-linked signaling was enriched in the suboptimal cases, whereas immunostimulatory interactions dominated in the optimal cases (Fig. 5b). Collectively, these data implicate CAF-derived ligands as upstream sustainers of resistant tumor programs and highlight stromal–epithelial interactions as candidate intervention points to overcome NCRT resistance.

### Spatial coupling of fibroblast–tumor crosstalk

**Fig. 6:**
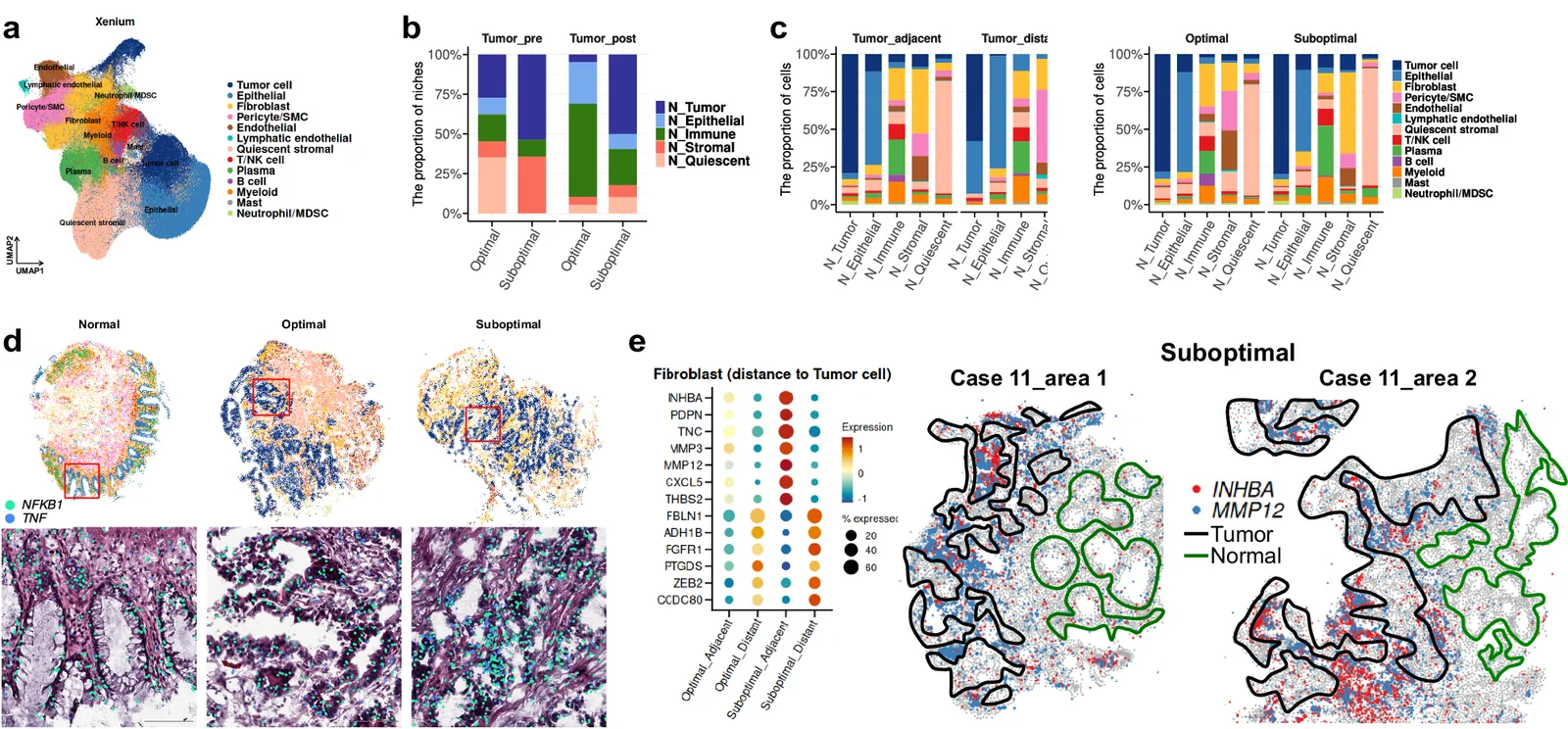
Spatial architecture of tumor-stroma niches in locally advanced rectal cancer. **a** Uniform manifold approximation and projection (UMAP) embedding of the Xenium spatial transcriptome dataset, colored by spatial clusters representing major epithelial, stromal, immune, and vascular compartments. **b** Stacked bar plots showing the distribution of five spatial niches across treatment and response groups. **c** Proportion of spatial clusters within each spatial niche, comparing tumor-adjacent versus tumor-distant regions (left) and optimal versus suboptimal responders (right). **d** Representative tissue sections annotated by spatial clusters (top) and corresponding magnified hematoxylin and eosin images overlaid with *NFKB1* and *TNF* expression (bottom), demonstrating differential inflammatory and stress-response signals across niches and treatment groups. **e** Dot plot showing the average expression of differentially expressed genes between tumor-adjacent and tumor-distant fibroblasts across response groups (left; values are *z*-score-transformed), alongside representative Xenium images displaying *INHBA* and *MMP12* expression patterns in two tumor areas from a suboptimal responder (right). Black and green contours indicate malignant and normal epithelial regions, respectively. MDSC, myeloid-derived suppressor cell; NK, natural killer; SMC, smooth muscle cell; TNF, tumor necrosis factor.

We analyzed the spatial architecture of LARC by integrating scRNA-seq references with spatial transcriptome, identifying discrete tissue clusters (Fig. 6a and Supplementary Fig. 7). They were further organized into five spatial niches defined by unique combinations of functionally relevant cellular neighborhoods: tumor, non-tumor epithelial, stromal, immune, and quiescent niches. The niche abundances differed by treatment states (pre-CRT and post-CRT) and response groups (Fig. 6b). Within the stromal niches, the fibroblast density was higher in tumor-adjacent areas than in tumor-distant areas, consistent with the role of stroma in shaping TME. Moreover, fibroblasts were significantly more abundant in tumor-adjacent regions of the suboptimal group compared with the optimal group, reinforcing stronger fibroblast–tumor coupling in the non-responders (Fig. 6c). NF-κB-related tumor regions denoted by *NFKB1* and *TNF* expressions were more prevalent in the suboptimal cases than in the optimal cases (Fig. 6d). Fibroblast localization further delineated distinct stromal states, revealing strong enrichment of mCAF-associated genes, including *INHBA*, *MMP12*, and *THBS2*, in tumor-adjacent fibroblasts, with the difference accentuated in the suboptimal group (Fig. 6e, left). Representative sections confirmed the colocalization of tumor-fibroblast clusters with *INHBA* and *MMP12* enrichment at tumor margins (Fig. 6e, right). Together, these data demonstrated a spatially anchored resistant ecosystem, characterized by *INHBA*⁺ fibroblast-rich stroma near NF-κB-high tumor cells, indicating that NCRT response was associated with dissolution of these niches.

### Immune signatures related to chemoradiation responsiveness

**Fig. 7:**
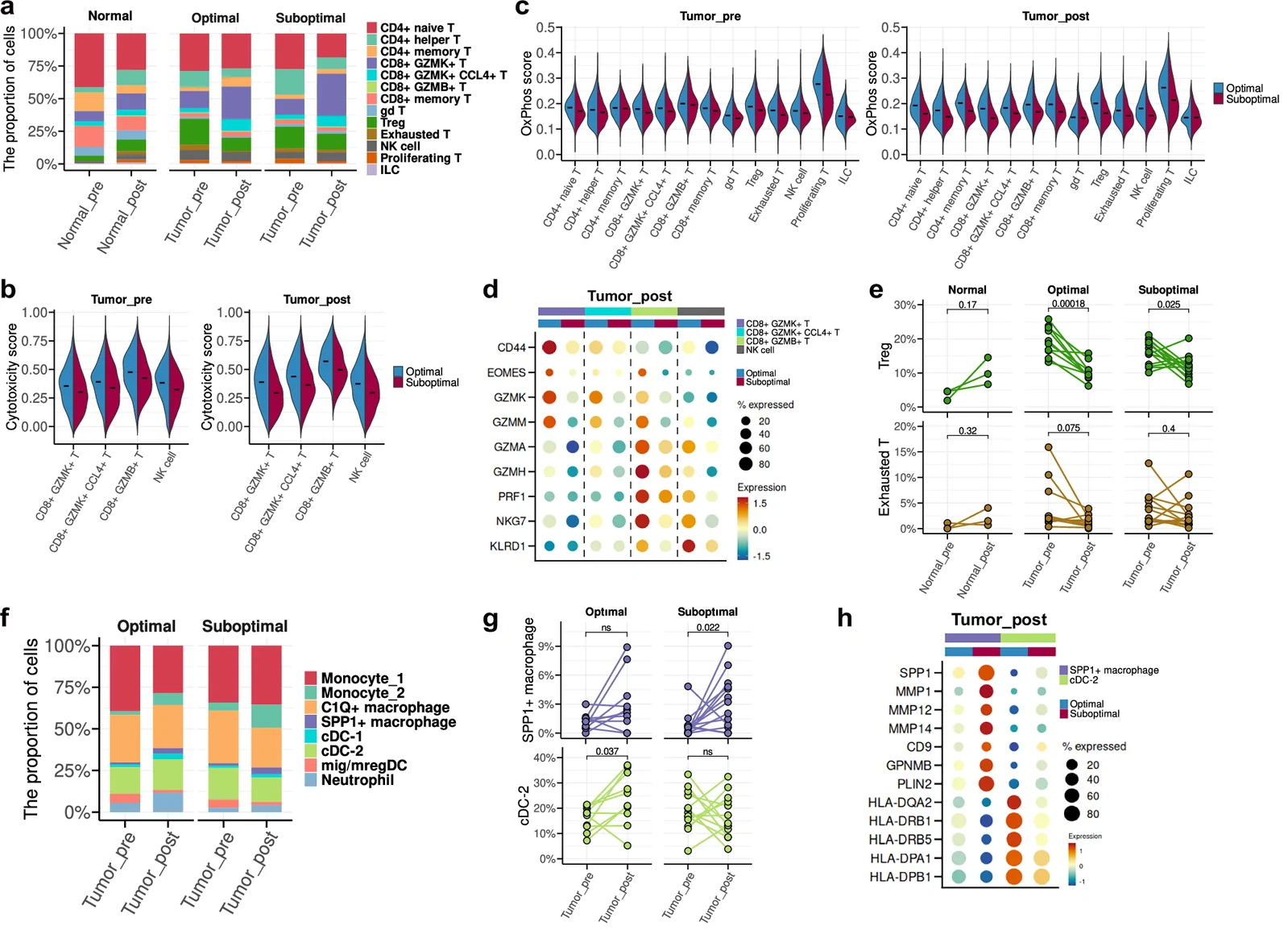
Immune subsets associated with therapeutic responses to neoadjuvant chemoradiotherapy. **a** Stacked bar plots showing the distribution of T/NK cell subclusters across groups. Violin plots depicting single-cell cytotoxicity scores (part **b**) and OxPhos scores (part **c**), with median values indicated by black crossbars. Statistical significance was assessed using Welch’s *t* test. **d** Dot plot showing the average expression of representative cytotoxic effector genes in CD8^+^ effector T cells and NK cells stratified by response groups. **e** Within-patient comparisons of the T_reg_ and exhausted T cell proportions across treatment and response groups. Each line connects paired samples from the same patients. Statistical significance was determined using paired *t* test. **f** Stacked bar plots illustrating the distribution of myeloid subclusters across groups. **g** Within-patient comparisons of SPP1⁺ macrophages and cDC-2 proportions across treatment and response groups. Each line connects paired samples from the same patients. Statistical significance was evaluated using paired *t* test. **h** Dot plot showing the average expression of representative SPP1⁺ macrophage-associated and antigen-presentation-associated genes in SPP1⁺ macrophage and cDC-2 subclusters across response groups. All values were *z*-score-transformed. cDC, conventional dendritic cell; NK, natural killer; ns, not significant; OxPhos, oxidative phosphorylation; T_reg_, regulatory T.

Subclustering of the T/NK cells in the scRNA-seq identified multiple subpopulations (Supplementary Fig. 2c and Fig. 7a). Among them, CD8^+^ effector and NK cells showed significantly higher cytotoxicity scores in the optimal group compared with the suboptimal group at both pre-CRT and post-CRT phases (Fig. 7b), implying that the optimal group experienced an induction of cytotoxic activity. Additionally, the OxPhos score was significantly higher in the optimal group across various T/NK subclusters (Fig. 7c). Cytotoxic effector genes, including *GZMK*, *GZMA*, *PRF1*, and *NKG7*, showed elevated expression levels in the optimal group (Fig. 7d). Additionally, paired longitudinal comparisons showed that both T_reg_ and exhausted T cell proportions tended to decrease more prominently in the optimal group after NCRT (Fig. 7e). Within the myeloid compartment (Supplementary Fig. 2f and Fig. 7f), the optimal group displayed increased conventional dendritic cell (cDC)-2 frequencies after therapy, whereas the suboptimal group exhibited a significant post-CRT increase in SPP1⁺ macrophages (Fig. 7g), consistent with established roles of SPP1⁺ macrophages in tumor-promoting and matrix-remodeling programs. Post-CRT transcriptional profiles further supported this divergence: cDC-2 from the optimal group was enriched for antigen-presentation signatures, whereas SPP1⁺ macrophages from the suboptimal group retained higher expressions of *SPP1*, MMPs, *CD9*, *GPNMB*, and *PLIN2* (Fig. 7h). These data indicated that NCRT responders underwent coordinated immune reprogramming, characterized by enhanced cytotoxic activity, increased OxPhos, contraction of regulatory/exhausted T cell states, and cDC-2 enrichment, whereas suboptimal cases retained a more suppressive immune landscape marked by increased SPP1⁺ macrophages.

## Discussion

Various biomarkers have been proposed to predict responses to NCRT in LARC^39–41^. However, no definite markers have yet been established as a reliable indicator for clinical application. Recently, TME-based markers, particularly those related to CAFs, immune cells, and ECM remodeling, have garnered increasing attention for low-to-intermediate-risk LARCs receiving neoadjuvant chemotherapy^22,42,43^. In this study, we used scRNA-seq to comprehensively profile both tumor and TME cell populations in intermediate-to-high-risk LARCs with NCRT. We newly demonstrated that combined NF-κB and OxPhos scores effectively discriminated between optimal and suboptimal groups (AUC = 0.875), suggesting potential molecular determinants predictive of the NCRT responsiveness in the intermediate-to-high-risk patients. Previous studies have indicated that high OxPhos activation^44^ and low NF-κB levels^45^ were associated with improved therapeutic responses in other cancers.

To identify mechanisms of resistance to NCRT in LARC, we compared molecular signatures between optimal and suboptimal response groups. Our analysis revealed that various cell types, including tumor cells, CAFs, and immune cells, interacted and communicated with one another. Notably, a specific subtype of tumor cells (*Tumor_2*) was significantly enriched in resistant tumors. *Tumor_2* displayed heightened activities of NF-κB, Wnt, Notch, VEGF, fatty acid, and glutamate metabolisms, when compared with the less-resistant *Tumor_1*. NF-κB activation promotes survival, DNA repair, and inflammatory gene expression, leading to chemotherapy and radiation therapy resistance in many cancers^46,47^. Similarly, activation of Wnt^48^, Notch^49^, VEGF^50^, fatty acid metabolism^51^, and glutamate metabolism^52^ is well known to contribute to resistance against chemotherapy or radiotherapy.

Multiple distinct subsets of CAFs have been identified, ranging from mCAF enriched with fibrosis and ECM genes, to inflammatory and vascular CAFs. They exhibit unique signaling interactions with both tumor and immune cells, indicating that CAF heterogeneity influences tumor behavior, immune exclusion, and response to therapy^38^. High mCAF activity is associated with a poor therapeutic prognosis in some cancers^53^; however, its contribution to NCRT resistance remains unclear in rectal cancers. Although the transcriptional features of mCAFs can overlap with wound-healing and fibrosis-associated responses, several observations support the view that these stromal states represent resistance-associated niches rather than diffuse fibrosis or structural scar-like phenotypes. In our study, mCAF levels decreased markedly after NCRT in patients who responded well but persisted in poor responses, indicating a potential link between mCAF and NCRT resistance. mCAF, enriched for ECM-remodeling and TGF-β-associated genes such as *INHBA*, *TGFBI*, and *MMP1*, maintained strong crosstalk with tumor cells through TGFB1–NF-κB signaling pathway^54^, reinforcing pro-fibrotic and inflammatory programs. Spatial and single-cell analyses revealed that enriched mCAFs were located near tumor areas with active NF-κB signaling, implying that mCAFs have a role in tumor promotion and resistance through ECM remodeling in rectal cancers. Furthermore, mCAF was correlated with the infiltration of exhausted and T_reg_ cells, suggesting their contribution to immunosuppression and NCRT resistance. Therefore, targeting the interactions among mCAFs, tumor cells, and T cells or focussing on the TGF-β/NF-κB pathways could enhance the effectiveness of NCRT in rectal cancer.

Our findings uncover a dynamic immune remodeling process closely linked to NCRT responsiveness in rectal cancer. In the optimal-response group, CD8⁺ effector T cells maintained strong cytotoxic and OxPhos activities, and cDC-2 myeloid cells displayed enhanced antigen-presenting capacity, reflecting effective immune activation following NCRT. By contrast, suboptimal responders exhibited pronounced T cell exhaustion and T_reg_ accumulation with reduced metabolic and cytolytic activity. Collectively, these findings indicate that combining CRT with immunomodulatory strategies, such as reinvigorating exhausted T cells and disrupting fibrotic, NF-κB-driven stromal barriers, could improve therapeutic responses in intermediate-to-high-risk rectal cancers receiving NCRT.

Interestingly, one patient (case 11) harbored synchronous double primary tumors located in the rectum and sigmoid colon, both displaying MSS phenotypes and canonical APC-driven mutation profiles. Despite their distinct anatomic origins and partially divergent mutational spectra (APC/FBXW7/AKT1 in the rectal and APC/TP53 in the sigmoid lesion), both tumors exhibited similarly suboptimal responses to NCRT and convergent transcriptional features characterized by NF-κB activation and mCAF enrichment. This consistency suggests that the resistant phenotype may be shaped less by site-specific or clonal genetic differences and more by shared adaptive responses within the epithelial–stromal ecosystem under CRT stress.

A recent study^22^ analyzed intermediate-to-low-risk LARCs treated with FOLFOX-based or FOLFOXIRI-based chemotherapy. The findings showed substantial CAF subtype remodeling, suggesting stromal reprogramming as a key driver of tumor progression and immunomodulation. In our cohort of intermediate-to-high-risk LARCs treated with capecitabine-based chemoradiation, we observed a transition in CAFs, marked by ECM-centered mCAF enrichment in the patients with poor responses. This was somewhat different from the epithelial–mesenchymal transition-centered negative CAF enrichment of the previous study^22^. In addition, our data revealed a distinct tumor-intrinsic reaction not seen in the chemotherapy-based study^22^: tumor cells following NCRT exhibited strong NF-κB activation, which was closely related to poor response and resistance. This mechanistic divergence between chemotherapy and CRT may reflect differences in risk groups; higher-risk tumors in our study displayed greater stress-adaptive and inflammatory signaling. Overall, our findings build on previous research by demonstrating CRT-specific reshaping of fibroblast populations and NF-κB-mediated adaptive programs in tumor cells. This highlights the dual remodeling of stromal and epithelial compartments in the outcomes of patients with higher-risk LARC receiving NCRT.

In conclusion, our study delineates comprehensive single-cell and spatial landscapes of higher-risk rectal cancers in response to NCRT, emphasizing the tripartite epithelial–stromal–immune remodeling as a determinant of therapeutic outcomes. The combined NF-κB and OxPhos scores, along with stromal and immune parameters, emerged as potential predictive biomarkers for stratifying patients based on therapeutic responsiveness. Targeting the reciprocal reinforcement between tumor-intrinsic NF-κB activation and stromal TGF-β-driven fibrosis, along with strategies to reinvigorate antitumor immunity, may therefore represent a promising approach to overcoming NCRT resistance and improving therapeutic efficacy in intermediate-to-high-risk LARCs.

## Supplementary information

## Data Availability

The data generated in this study are available upon request from the corresponding author.
