# Supplementary material for: NF-κB-active tumors with matrix CAFs and suppressive immunity as key resistance mechanisms to chemoradiation in rectal cancer
Source: Exp Mol Med. 2026 Aug 4;58(8):2643–53. doi: 10.1038/s12276-026-01792-2 (PMC13538494; doi:10.1038/s12276-026-01792-2)
Supplement: Supplementary file 1 [file 12276_2026_1792_MOESM1_ESM.pdf]

Supplementary Information

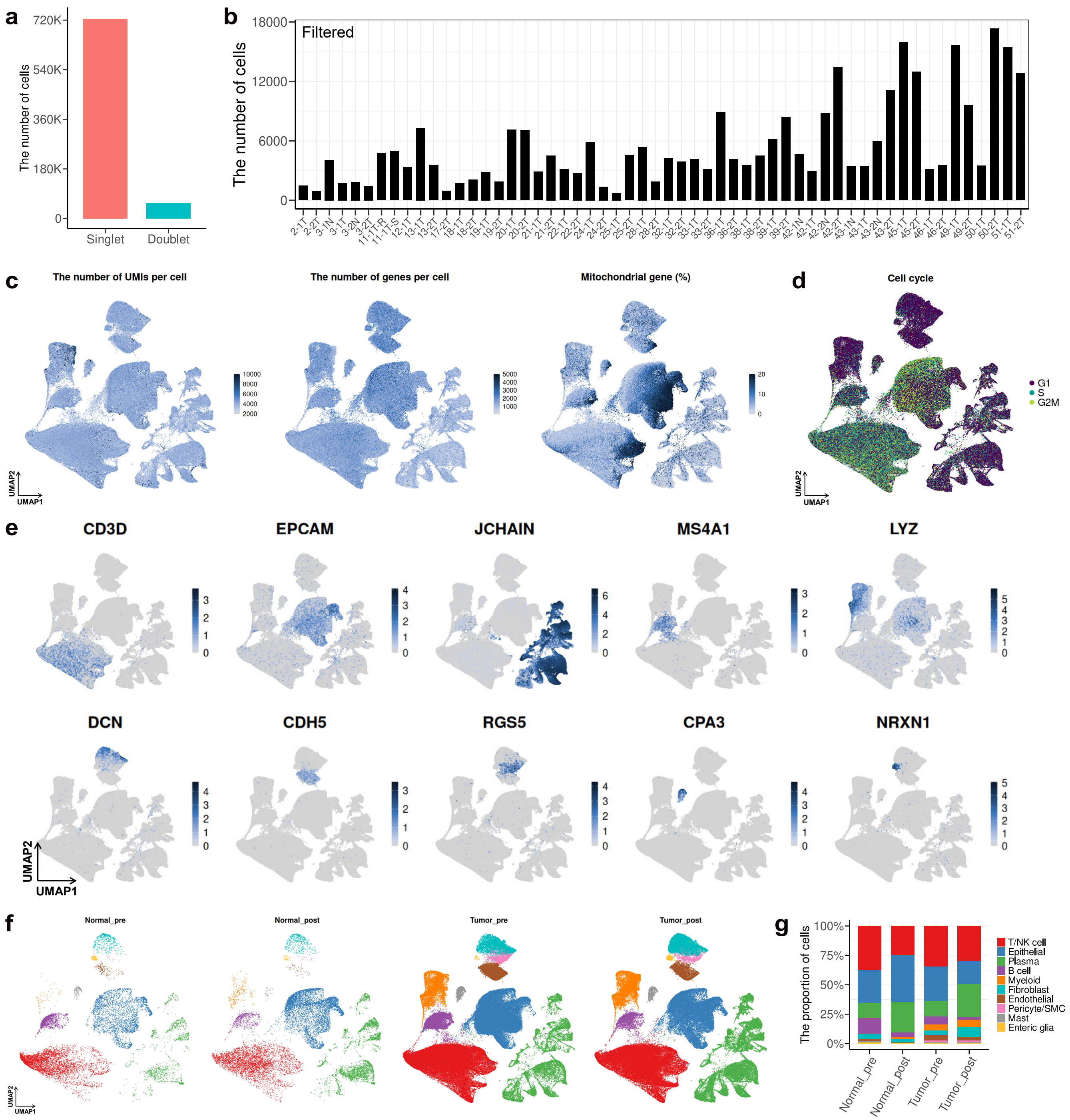

Supplementary Fig. S1. Quality control and overview of scRNA-seq data. a The number of predicted singlets and doublets inferred using DoubletFinder. **b** Cell counts retained per sample after quality-control filtering. **c** UMAP embeddings colored by total UMI counts (left), the number of detected genes (center), and mitochondrial RNA fraction (right). **d** UMAP visualization colored by inferred cell-cycle phase. **e** UMAP plots showing the expression of canonical marker genes used for cluster annotation. **f**, **g** UMAP embeddings (**f**) and corresponding cell-type composition (**g**) stratified by pathology and treatment groups.

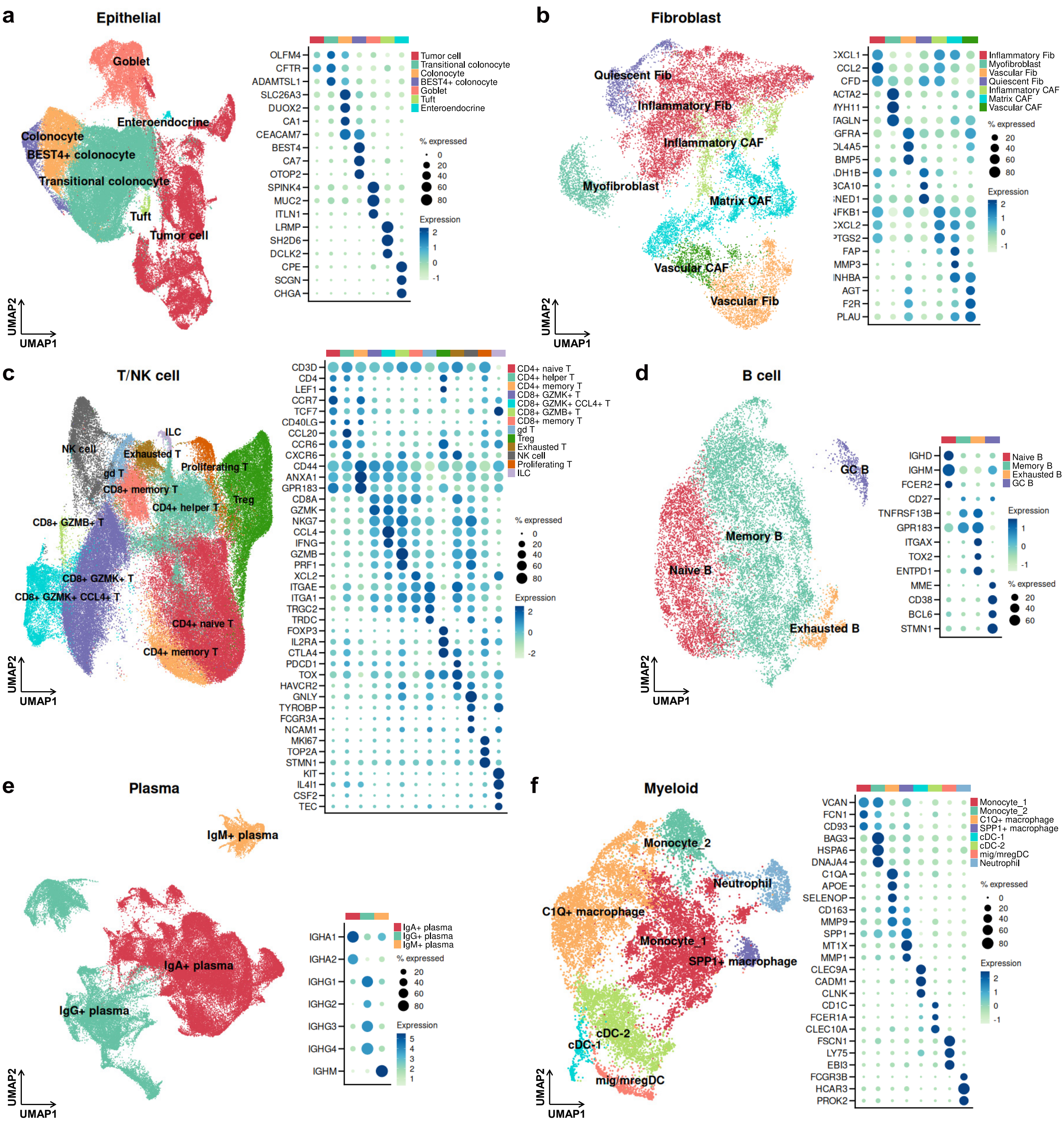

Supplementary Fig. S2. Subclustering of major cell compartments. UMAP embeddings of subclusters (left) and corresponding dot plots of marker genes (right) for the epithelial (**a**), fibroblast (**b**), T/NK cell (**c**), B cell (**d**), plasma (**e**), and myeloid (**f**) compartments. Dot size indicates the fraction of cells expressing each marker, and color reflects the average expression levels.

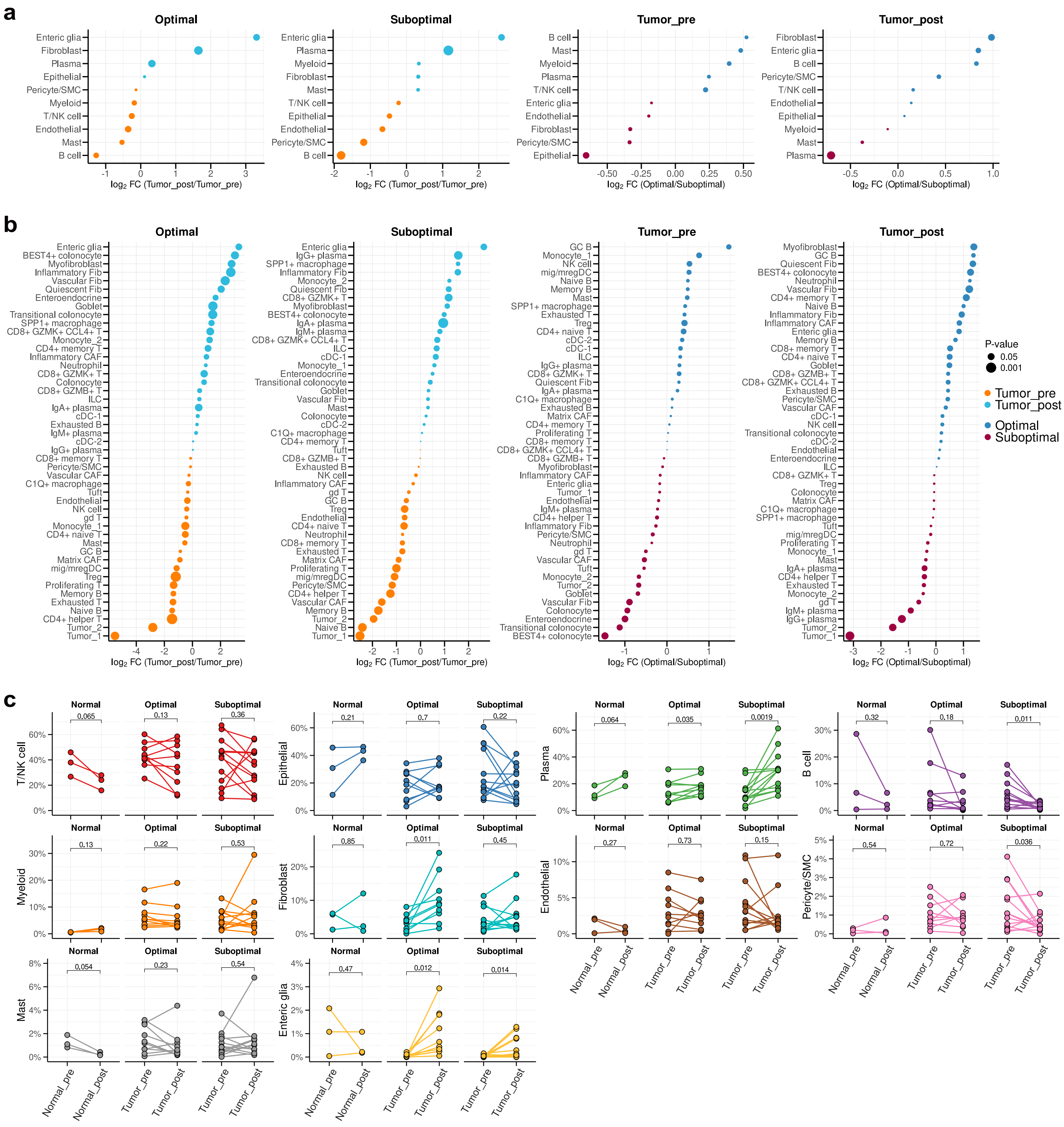

Supplementary Fig. S3. Differential abundance across treatment and response groups. Bubble plots illustrating changes in major clusters (**a**) and subclusters (**b**). The x-axis represents $\log_2$ fold change, and bubble size denotes statistical confidence $-\log_{10}(P\text{-value})$ determined by Welch's *t*-test. Positive x-values indicate enrichment after NCRT or in the optimal-response group. **c** Patient-level paired changes in proportions of major cell lineages according to treatment status and response groups. Each line connects paired samples from the same patients. Statistical significance was assessed using paired *t*-test.

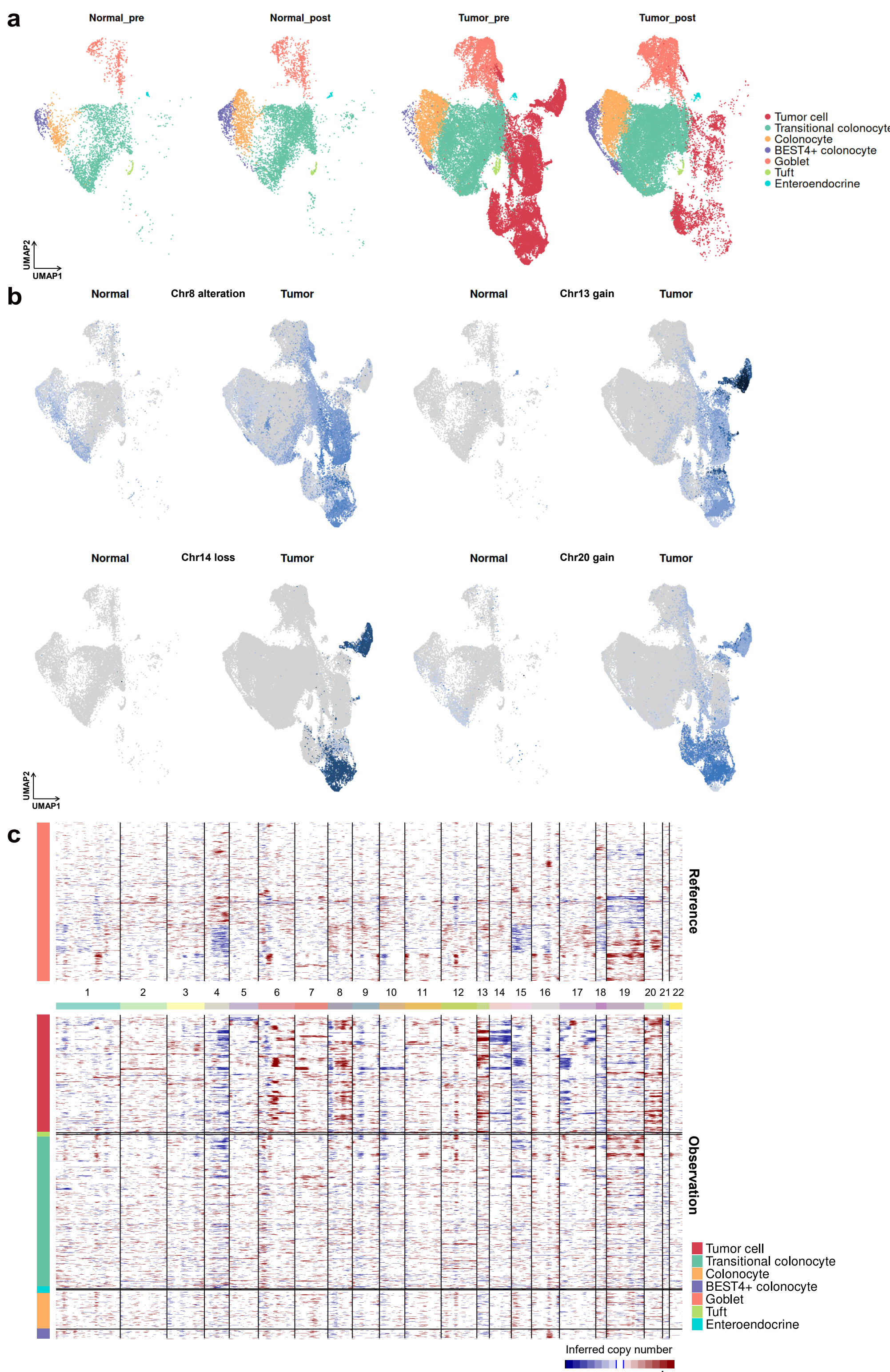

Supplementary Fig. S4. Identification of malignant epithelial cells by inferred CNAs. a UMAP plots of epithelial cells shown separately for each group. **b** UMAP projection of inferred CNA profiles for chromosomes 8, 13, 14, and 20. **c** Heatmap of cell-by-genome inferred CNA profiles relative to goblet cells (diploid reference). Rows represent individual cells, and columns denote ordered genomic bins. The color scale reflects relative copy-number gains and losses.

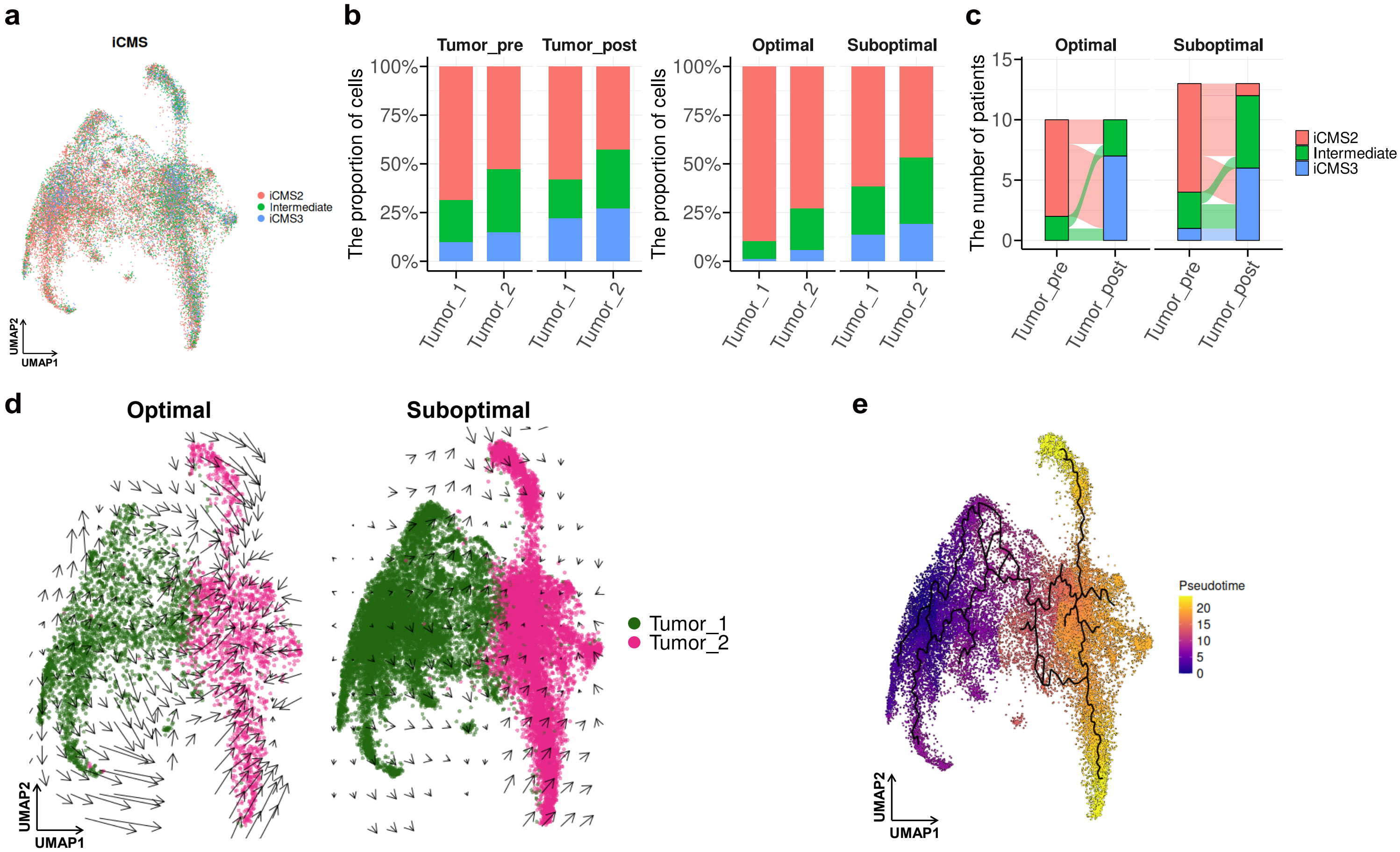

Supplementary Fig. S5. iCMS annotation and dynamic inference of tumor subclusters. a UMAP plots of tumor cells colored by iCMS subtypes. **b** Stacked bar plots showing per-cell relative proportions of iCMS states across tumor subclusters, stratified by treatment (left) and response (right) groups. **c** Sankey plot showing per-patient relative proportions of iCMS states across treatment and response groups. **d** RNA velocity projected onto the tumor UMAP across response groups. **e** Pseudotime trajectory projected onto the tumor UMAP.

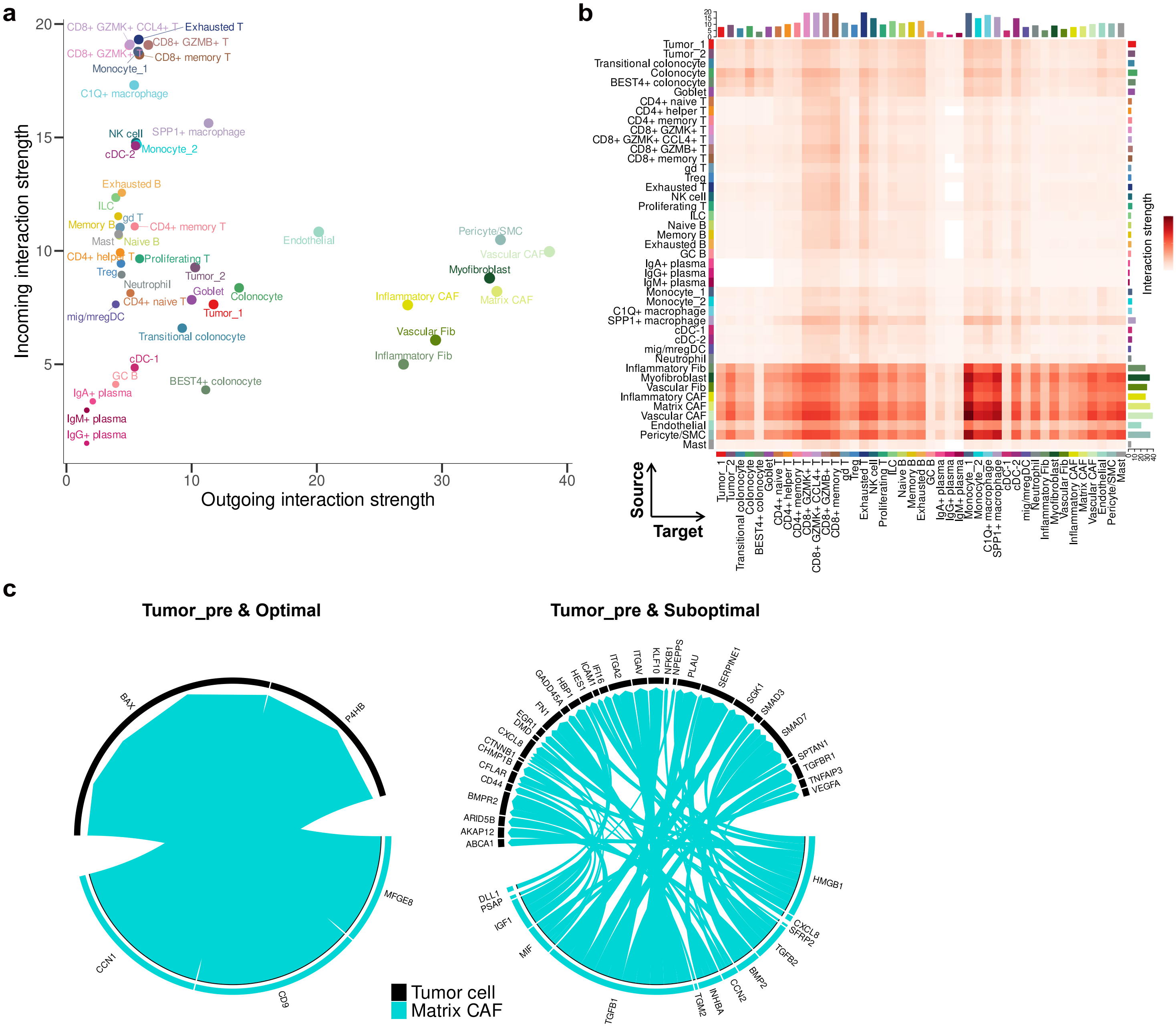

Supplementary Fig. S6. Overview of intercellular communication networks. Intercellular interactions inferred by CellChat for subclusters containing > 20 cells across all groups. **a** Scatter plot depicting total outgoing (x-axis) versus incoming (y-axis) communication probability aggregated for each subcluster. Dot size corresponds to the number of inferred ligand-receptor interactions. **b** Heatmap illustrating interaction strength among cell populations. **c** Circle plots illustrating inferred communication from ligands of mCAFs to target genes in tumor cells across response groups at baseline.

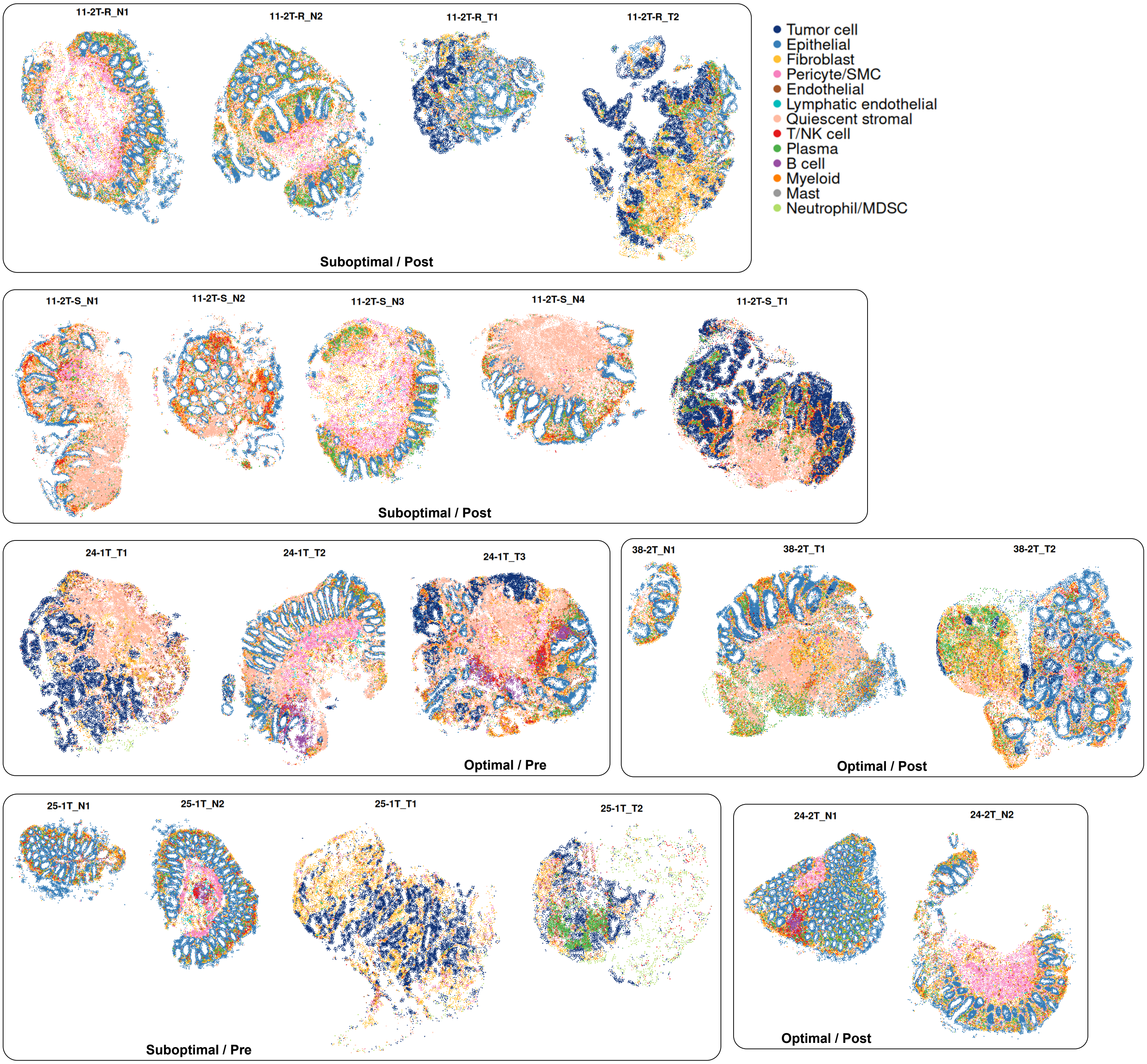

Supplementary Fig. S7. Spatial clustering across all regions of interest. Tissue images colored by spatial clusters for all dissected regions of interest.

Supplementary Table S1. Clinical characteristics of patients included in this study.

Patient ID	Sex	Age	Clinical stage (TNM)	Tumor location in rectum	Risk category	Sampling site	NCRT	Tumor regression grade (0-3)	Response group	scRNA-seq	Spatial-seq
2	M	74	cT3N1M0 (III)	middle	intermediate	Tumor	Pre	2	Suboptimal	v	
						Tumor	Post			v	
3	M	42	cT3N2M0 (III)	middle	high	Tumor	Pre	0	Optimal	v	
						Tumor	Post			v	
						Normal	Pre			v	
						Normal	Post			v	
11	F	80	cT3N2M0 (III)	lower	intermediate	Tumor_R	Pre	2	Suboptimal	v	
						Tumor_R	Post				v
			cT3N2M0 (III)			Tumor_S	Pre	2	Suboptimal	v	
						Tumor_S	Post				v
12	F	78	cT3N2M0 (III)	middle	intermediate	Tumor	Pre	2	Suboptimal	v	
13	M	66	cT3N1M0 (III)	middle	intermediate	Tumor	Pre	2	Suboptimal	v	
						Tumor	Post			v	
17	M	48	cT3N1M0 (III)	lower	high	Tumor	Post	2	Suboptimal	v	
18	M	66	cT3N1M0 (III)	middle	intermediate	Tumor	Pre	2	Suboptimal	v	
						Tumor	Post			v	
19	M	56	cT3N2M0 (III)	lower	intermediate	Tumor	Pre	1	Optimal	v	
						Tumor	Post			v	
20	M	86	cT3N2M0 (III)	middle	intermediate	Tumor	Pre	1	Optimal	v	
						Tumor	Post			v	
21	F	60	cT3N0M0 (II)	middle	intermediate	Tumor	Pre	0	Optimal	v	
						Tumor	Post			v	
22	M	67	cT3N2M0 (III)	middle	high	Tumor	Pre	2	Suboptimal	v	
						Tumor	Post			v	
24	M	64	cT3N2M0 (III)	lower	high	Tumor	Pre	1	Optimal	v	v
						Tumor	Post			v	v
25	M	58	cT3N1M0 (III)	lower	high	Tumor	Pre	2	Suboptimal	v	v
						Tumor	Post			v	
28	M	48	cT3N2M0 (III)	middle	intermediate	Tumor	Pre	0	Optimal	v	
						Tumor	Post			v	
32	M	66	cT3N2M0 (III)	middle	intermediate	Tumor	Pre	0	Optimal	v	
						Tumor	Post			v	
33	M	82	cT3N2M0 (III)	middle	intermediate	Tumor	Pre	2	Suboptimal	v	
						Tumor	Post			v	

36	M	51	cT3N2M0 (III)	lower	intermediate	Tumor	Pre	2	Suboptimal	v	
						Tumor	Post			v	
38	F	77	cT3N0M0 (II)	middle	intermediate	Tumor	Pre	0	Optimal	v	
						Tumor	Post			v	v
39	M	53	cT3N2M0 (III)	lower	high	Tumor	Pre	2	Suboptimal	v	
						Tumor	Post			v	
42	M	61	cT3N2M0 (III)	lower	high	Tumor	Pre	2	Suboptimal	v	
						Tumor	Post			v	
						Normal	Pre			v	
						Normal	Post			v	
43	M	61	cT3N2M0 (III)	middle	high	Tumor	Pre	2	Suboptimal	v	
						Tumor	Post			v	
						Normal	Pre			v	
						Normal	Post			v	
45	M	60	cT3N2M0 (III)	lower	intermediate	Tumor	Pre	2	Suboptimal	v	
						Tumor	Post			v	
46	M	59	cT3N2M0 (III)	middle	intermediate	Tumor	Pre	0	Optimal	v	
						Tumor	Post			v	
49	F	49	cT3N2M0 (III)	middle	intermediate	Tumor	Pre	2	Suboptimal	v	
						Tumor	Post			v	
50	F	47	cT3N1M0 (III)	middle	intermediate	Tumor	Pre	0	Optimal	v	
						Tumor	Post			v	
51	M	57	cT3N2M0 (III)	middle	intermediate	Tumor	Pre	3	Suboptimal	v	
						Tumor	Post			v	

Supplementary Table S2. Gene panel for Xenium in situ transcriptomics.

ABCC11	CCN2	COL8A1	ERCC2	HLA-A	KLRD1	MYC	SARAF	TNC
ACTA2	CCND1	CPA3	EREG	HLA-DRA	KLRF1	MYL9	SAV1	TNF
ADH1B	CCND2	CSF1	ERF	HOPX	KLRK1	MYLK	SELL	TNFAIP6
ADIPOQ	CCNE1	CSF1R	ETV1	HPGDS	KRAS	NCAM1	SELP	TNFRSF13B
ADORA2A	CCR2	CSF3R	EZH2	HRAS	KRT13	NCR1	SEMA3C	TNFRSF13C
AGO2	CCR5	CST7	FABP5	ICAM1	KRT14	NELL2	SERPINA3	TNFRSF17
AGR2	CCR7	CTLA4	FAP	ICOS	KRT18	NFKB1	SERPINB3	TNFRSF18
AIFM2	CD14	CTNNB1	FAS	ICOSLG	KRT19	NKG7	SERPINF1	TNFRSF1B
AIM2	CD163	CTSC	FASLG	IDO1	KRT4	NKX2-1	SFRP4	TNFRSF9
AKR1C3	CD1C	CTSD	FASN	IFITM1	KRT7	NOSIP	SIGLEC10	TNFSF12
AKT1	CD1E	CTSS	FBLN1	IFNG	KRT8	NOTCH1	SIRPA	TNFSF8
ALDH1L1	CD2	CTSW	FCER1A	IGF1	KYNU	NOTCH2	SLAMF7	TNFSF9
ALK	CD247	CTTN	FCER1G	IGFBP3	LAG3	NOX4	SLC39A6	TOP2A
ANGPT1	CD27	CX3CR1	FCER2	IGHA2	LAMP3	NTRK1	SMAP2	TOX2
ANXA1	CD274	CXCL1	FCGR3A	IGHD	LATS1	NTRK2	SOD2	TP53
APC	CD276	CXCL10	FCGR3B	IGHM	LATS2	PAK1	SORL1	TP63
APCDD1	CD38	CXCL11	FCRL1	IKBKE	LDHB	PAX5	SOX17	TPD52
APOBEC3A	CD3D	CXCL12	FCRL3	IKZF2	LEF1	PCLAF	SOX2	TPM1
APOC1	CD3E	CXCL13	FGF1	IL10	LGMN	PDCD1	SOX4	TPM2
APOE	CD3G	CXCL14	FGF18	IL11	LIF	PDCD1LG2	SPINK1	TPSAB1
AQP1	CD4	CXCL16	FGF19	IL12A	LILRA4	PDGFA	SPOCK2	TPT1
AQP3	CD40	CXCL2	FGFBP2	IL13	LILRA5	PDGFRA	SPON2	TRAC
AR	CD40LG	CXCL3	FGFR1	IL17A	LILRB2	PDGFRB	SPP1	TRAT1
ARG1	CD44	CXCL5	FGFR2	IL17RB	LRRC15	PDK1	SRC	TRBC2
ATM	CD47	CXCL6	FGFR3	IL1A	LTB	PDPN	SSPN	TRDC
AURKA	CD68	CXCL9	FGFR4	IL1B	LUM	PECAM1	STAT3	TREM1
AXIN1	CD69	CXCR2	FHIT	IL2	LY6G6C	PIK3CA	STC1	TREM2
AXL	CD70	CXCR4	FHL2	IL23A	LYVE1	PIM1	STK3	TSC1
BANK1	CD79A	CXCR6	FLNB	IL2RA	MAPK1	POSTN	STK4	TSC2
BCL2	CD80	CYTIP	FLT3	IL2RB	MAX	POU2AF1	SYTL1	TSPAN32
BCL6	CD86	DCN	FN1	IL32	MCAM	PRDM1	TAC1	TUBA4A
BRAF	CD8A	DDR2	FOLR1	IL4	MDM2	PRDX2	TACSTD2	TYMP
BRD4	CD8B	DGKA	FOLR2	IL4R	MERTK	PRF1	TAGLN	TYROBP
BTLA	CD96	DMKN	FOXA1	IL6	MET	PROX1	TC2N	VAV2
BTN3A1	CDH1	DNMT3B	FOXP3	IL7R	MGA	PTEN	TCEAL7	VCAN
C1QA	CDK8	DPT	FYN	INHBA	MICA	PTGDS	TCF7	VEGFA
C1QB	CDKN1A	DST	G0S2	ISG15	MITF	PTK2	TCF7L1	VEGFD

C1QC	CEACAM6	DUOX1	GATA3	ITGA1	MKI67	PTPRC	TENT5C	VIM
CARD11	CEACAM8	DUSP2	GLI1	ITGA4	MMP11	RAC1	TERT	VSIG4
CAV1	CENPF	E2F3	GNLY	ITGAE	MMP12	RAF1	TEX9	VSIR
CCDC80	CHEK2	EDN1	GPR183	ITGAV	MMP14	RB1	TFF3	VTCN1
CCL13	CLDN4	EDNRB	GPRC5A	ITGB1	MMP19	RHEB	TGFB1	VWF
CCL17	CLDN6	EGF	GPX2	ITGB2	MMP2	RHOA	TGFBR1	WARS
CCL18	CLEC10A	EGFR	GSK3B	ITGB6	MMP3	RHOH	THBS1	WDFY4
CCL19	CLEC3B	EIF4EBP1	GZMA	ITK	MMP9	RORC	THBS2	WNT1
CCL2	CLEC4E	ELANE	GZMB	ITM2A	MRC1	ROS1	THY1	WNT7B
CCL20	CLEC7A	ELF3	GZMH	ITM2C	MS4A1	RTKN2	TIFA	WT1
CCL21	CLEC9A	ENTPD1	GZMK	JAG1	MS4A2	RUNX1	TIGIT	XBP1
CCL22	CLIC3	EOMES	HAVCR2	JUN	MS4A6A	S100A12	TLR4	XCL1
CCL24	COCH	EPCAM	HCST	KDR	MSR1	S100A4	TLR9	ZEB1
CCL4	COL14A1	EPHB1	HES1	KIT	MTOR	S100A6	TMC5	ZEB2
CCL5	COL15A1	ERBB2	HEY1	KLRB1	MUC6	S100A8	TMSB10	ZNF683
CCL8	COL1A2	ERBB3	HGF	KLRC1	MYBPC1	S100A9		
